# Plasma-modified filters improve recovery of viable fungal spores for culture and DNA analysis

**DOI:** 10.1007/s00253-026-14006-6

**Published:** 2026-08-14

**Authors:** Hafiza Umaima Affan, Claire Lenehan, Sally Fryar, Michael Taylor, Melanie MacGregor

**Affiliations:** 1 College of Science and Engineering, Flinders University, Sturt Road, Bedford Park, South Australia 5042 Australia; 2 Flinders Institute for Nanoscale Science and Technology, Flinders University, Sturt Road, Bedford Park, South Australia 5042 Australia; 3 WSP Australia Pty Limited, Adelaide, South Australia 5000 Australia

**Keywords:** Plasma-coated filters, Fungal bioaerosols, Spore viability, DNA sequencing, PCR, Optimization parameters, Antifungal surface effect

## Abstract

**Abstract:**

This proof-of-concept study evaluates plasma-modified polyethylene terephthalate (PET) filters for fungal bioaerosol collection and downstream culture and molecular analyses. Filters were plasma-coated using either 2-methyl-2-oxazoline (POX) or perfluorooctane (PFO), to enhance fungal spore adhesion and recovery using *Aspergillus niger* and *Rhodotorula glutinis* as representative of filamentous and yeast-like fungal species, respectively. Optimal capture was achieved at 15 L/min for 30 min, with saturation reached at 38 mg using biomass from four to five colony plates. Viability tests confirmed that spores collected on PFO and non-coated (NC) filters retained the ability to germinate, whereas POX coatings inhibited regrowth, indicating an antifungal surface effect. Molecular analyses demonstrated that 14–16 mg of captured biomass yielded sufficient DNA for downstream analyses. The internal transcribed spacer (ITS) region amplified using PCR produced clear amplicons (~ 550 base pair (bp)) across all tested species. Sequencing provided accurate species identification with 99–100% similarity to GenBank reference sequences, verifying that plasma coatings did not compromise DNA quality, amplification efficiency, or sequencing integrity. These findings establish plasma-polymer-coated PET filters as effective and cultivation- and PCR-compatible tools for fungal bioaerosol collection and define practical operating parameters for their use in environmental assessments.

**Key points:**

*• Plasma-coated PET filters support integrated fungal bioaerosol analysis.*

*• PFO-coated filters enabled recovery of viable fungal spores.*

*• Recovered fungal biomass was suitable for PCR and ITS sequencing.*

**Graphical Abstract:**

**Figure:**
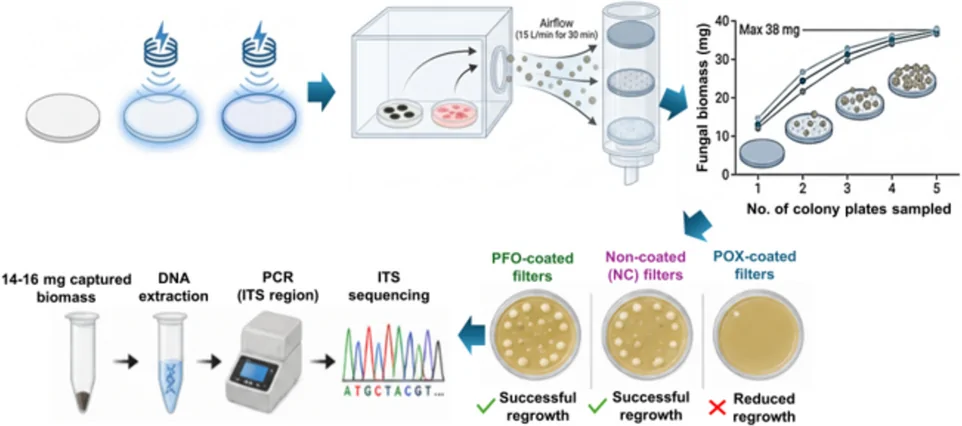

**Supplementary Information:**

The online version contains supplementary material available at https://doi.org/10.1007/s00253-026-14006-6.

## Introduction

Airborne fungal spores are a biologically complex component of atmospheric particulate matter. In indoor and outdoor environments, fungal bioaerosols contribute substantially to allergic diseases, respiratory illness, and exposure to opportunistic pathogens, while also playing a key role in ecosystem dispersal and microbial biogeography (Eduard 2009; Ferguson et al. 2019; Ghosh et al. 2015). Consequently, air sampling has become an essential tool in environmental mycology, occupational hygiene, and indoor air quality assessment. However, the analytical value of air samples depends not only on how many biological particles are physically captured, but also on how much of the recovered material remains biologically usable for downstream cultivation and molecular analyses.

Modern fungal surveillance increasingly relies on DNA-based methods, particularly sequencing of the internal transcribed spacer (ITS) region, which provides species-level resolution across the broadest range of the fungal kingdom (Schoch et al. 2012). For airborne fungi, this introduces a critical bottleneck from two distinct reasons. Firstly, the amount of biomass collected from air is dependent on the amount of biomass present, which varies between environments, and is often extremely small. Collection methods with high flow rates or high collection efficiencies are therefore necessary. In previous work, we demonstrated that plasma-modified filters could enhance fungal spore capture efficiency and are therefore an alternative worth exploring (Affan et al. 2026; Morgan et al. 2010). Secondly, fungal spores are among the most recalcitrant microbial structures from which to extract DNA. Their thick, melanized cell walls and protective surface proteins reduce lysis efficiency and DNA release, leading to stochastic PCR failure when starting material is limited (Mbareche et al. 2019; Rittenour et al. 2012). Moreover, nucleic acid yield, and hence microbial diversity recovered, depends on the extraction protocol used (Luhung et al. 2015; Morgan et al. 2010). Thus, even when spores are successfully captured on a filter, there is no guarantee that the recovered biomass will be sufficient for reliable molecular analysis. The challenge is further compounded by the physical and chemical environment of air-sampling filters. Filters can adsorb DNA, trap inhibitors, or retain spores in ways that reduce extraction efficiency, creating a disconnect between captured biomass and amplifiable DNA. Air filtration studies have documented significant variability in DNA yield depending on filter material and recovery protocols, suggesting that many filters retain biomass in a form that is difficult to lyse or recover for molecular analysis (Luhung et al. 2015; Morgan et al. 2010). Additionally, prolonged sampling has been associated with reduced DNA recoverability across different filter types, likely resulting from adsorption, degradation, or inhibitory filter interactions (Burton et al. 2005; Ferguson et al. 2019; Luhung et al. 2021, 2015). This shows that sampling conditions also play a decisive role in determining how much biologically useful material is obtained. Environmental airflow (e.g., windspeed/air movement) governs both aerosolization of spores and their deposition on filters, while sampling duration and spore loading affect whether sufficient biomass accumulates to reach molecular detection thresholds (Durand et al. 2002; Lin and Li 1998).

Building on our pioneering work demonstrating the promise of plasma-coated filters for high-efficiency fungal spore capture, this study evaluates their suitability for the next stage of application as integrated components of a complete biological and molecular analysis pipeline, from high efficiency collection through to spore recovery, viability assessment, and DNA extraction. To achieve this, sampling parameters including airflow rate, sampling duration, and saturation behavior were first optimized using two representative species, *Aspergillus niger* and *Rhodotorula glutinis*, selected to reflect filamentous and yeast-like fungi with contrasting surface properties. Following optimization, spores from four fungal species (*A. niger*, *Cladosporium* sp., *Penicillium roqueforti*, and *R. glutinis*) captured on plasma-coated filters were used to evaluate the viability of spores recovered from filters and to assess their compatibility for culture-based analysis. The minimum fungal biomass required for DNA extraction and PCR amplification was also established. The latter molecular analyses tested whether these plasma coatings interfere with the genomic material or cause PCR inhibition.

## Materials and methods

### Filter preparation by plasma polymerization

Synthetic heating, ventilation, and air conditioning (HVAC) filter material was obtained from RS Components, NSW, Australia, with the following specifications: pad filter, grade G4, and 20 mm nominal depth (RS Stock number 122–1774). The material was cut into 19 mm diameter in size before use. The filter fibers were composed of polyethylene terephthalate (PET) and arranged into a gauze-like material. The filter’s geometry results in nonuniform pore sizes, generally ranging from 5 to 15 μm, through which air passes between the fibers. Silicon wafers (D&X Co., Ltd, Tokyo, Japan) were purchased from Australian National Fabrication Facility (ANFF-SA, Adelaide, South Australia) and used as a reference material. Two monomers, purchased from Sigma-Aldrich, St. Louis, MO, USA, and Merck Life Science, Truganina, Australia, were used as precursors for plasma polymer deposition, namely 2-methyl-2-oxazoline (POX) and perfluorooctane (PFO).

Plasma polymer coatings were deposited onto PET filters and silicon wafers using a custom-made plasma reactor, details of which have been previously outlined (Chan et al. 2020). Briefly, the plasma was ignited by using 13.56-MHz radio frequency (RF) generator coupled to a matching network (Coaxial Power Systems, Eastbourne, UK). Before the plasma deposition, the sample substrates, including filters and silicon wafers, were placed on the plasma reactor’s lower electrode and primed at 30 W for 3 min with air plasma at 1.2 × 10^−1^ mbar. The reactor was then pumped down to a base pressure of 2 × 10^−2^ mbar. The pressure in the plasma chamber was monitored using a Pirani gauge (APG100, Edwards Vacuum, Burgess Hill, UK) and controlled by an air inlet needle valve (Chell Instrument, North Walsham, UK). For the plasma polymer deposition, two monomers POX and PFO were introduced into the plasma chamber at an ignition pressure of 0.12 and 0.20 mbar, respectively, following established procedures (Delcheva et al. 2024; MacGregor et al. 2019; Macgregor and Vasilev 2019). The deposition time was 90 s for POX to produce coatings 16 nm in thickness. For the PFO coatings, the monomer was deposited for 40 s to achieve the same thickness as POX. These coatings were chosen based on our previous findings which showed that they captured more fungal spores than other common plasma polymer coatings (Affan et al. 2026). Non-coated (NC) PET filters were used as the control substrate. As the objective of this study was to evaluate the effect of plasma-polymerized POX and PFO nanometre thin coatings on spore capture performance, comparisons were made between coated filters and the unmodified PET filter membrane rather than with conventionally deposited (non-plasma) polymer coatings.

### Fungal colonies culture

Four fungal species were used in this study: *Cladosporium* sp. (Environmental Health Lab, Flinders University), *A. niger* (ATCC 6257), *P. roqueforti* Thom (ATCC 10110), and *R. glutinis* (Fresenius) Harrison (ATCC 32765) obtained from American Type Culture Collection, Manassas, VA, USA. Growth media included malt extract agar (MEA, CM0059B) and potato dextrose agar (PDA, CM01390) from Thermo Fisher Scientific, Adelaide, Australia, and yeast malt agar (YM Agar, Y3127) from Merck Life Science, Truganina, Australia.

For media preparation, 50 g MEA, 39 g PDA, or 41 g YM agar were each dissolved in 1 L of deionized water, autoclaved at 121 °C for 15 min (fluid cycle), cooled, and poured into Petri dishes. Plates were left overnight in a biosafety cabinet to prevent condensation. *A. niger* and *Cladosporium* sp. were cultured on MEA, *P. roqueforti* on PDA, and *R. glutinis* on YM agar. Incubation was carried out at 26 °C for 5–7 days (*A. niger*, *P. roqueforti*, *R. glutinis*) and 18–20 days (*Cladosporium* sp.) to obtain mature sporulating colonies.

### Optimization of sampling conditions

Sampling conditions were optimized using a custom-made aerosolization sampling chamber described in detail in previous works (Affan et al. 2026). Briefly, in a closed plastic container, nitrogen is flown over fungal colonies grown in Petri dishes towards filters through which air is allowed to exit the chamber. Two fungal species with distinct morphologies and surface properties were selected for optimization of sampling conditions: *A. niger* (a filamentous species) and *R. glutinis* (a yeast species). As characterized in our previous study (Affan et al. 2026), *A. niger* spores were spherical to slightly elliptical, 3–5 µm in diameter, dark brown to black, and had a rough, echinulate surface. In comparison, *R. glutinis* spores (cells) were ovoid to globose, approximately 2–5 × 4–10 µm in size. Water-droplet tests performed on mature colonies indicated contrasting apparent wettability: water droplets remained rounded on the *A. niger* colony, indicating a hydrophobic colony surface, whereas they spread over the *R. glutinis* colony, indicating a hydrophilic colony surface. These observations were consistent with the surface chemistry identified by attenuated total reflectance-Fourier transform infrared spectroscopy (ATR-FTIR). Differences in size, morphology, surface texture, and apparent wettability may influence wetting and adhesion to the collection surface and may consequently affect capture efficiency. However, these species were chosen to assess how spore characteristics influence capture efficiency under different sampling parameters, namely airflow rate, time, and saturation.

Three types of PET filters were evaluated in triplicate for each sampling parameter: non-coated (NC), plasma-coated POX, and PFO filters. Filters were weighed in pre-weighed 15-mL centrifuge tubes before each sampling test. Following each sampling event, filters were carefully removed and transferred to pre-weighed 15-mL centrifuge tubes for spore recovery and mass quantification.

Captured spores were subsequently detached by fully submerging each filter in 4 mL of 1X phosphate-buffered saline (PBS) and vortexing at 2500 rpm for 5 min. 1X PBS was prepared from a 20X stock by mixing 50 mL of 20X PBS (POCD HealthCare, Macquarie Park, Australia) with 950 mL of deionized water to obtain 1 L of 1X PBS. The filters were then removed, and the spore suspensions were centrifuged at 4500 rpm for 5 min to pellet the spores. The supernatant was discarded, and tubes were dried overnight in a biosafety cabinet at room temperature to remove residual moisture. Dry spore pellets were quantified gravimetrically using an analytical balance (precision ± 0.1 mg) by subtracting the initial tube weight from the final dried weight. The used filters were also dried completely inside the biosafety cabinet at room temperature and observed under a Nikon ECLIPSE Ti2 Inverted research microscope (Tokyo, Japan) to ensure complete removal of spores.

#### Airflow rate

A preliminary set of experiments was conducted to evaluate how varying the flow rate influences the collection efficiency of fungal spores collected on different filter types. Three nominal flow rates were tested: 5 L/min, 10 L/min, and 15 L/min. Based on the British Oxygen Company (BOC, Windlesham, UK) nitrogen conversion factor (1.19), these corresponded to approximate effective nitrogen flow rates of 6, 12, and 18 L/min, respectively. The latter was the maximum stable flow rate the system could achieve without causing pressure instability or compromising filter sealing. Therefore, higher flow rates could not be tested. The sampling duration was kept constant at 30 min for all runs to ensure comparability across conditions. Collection time was investigated in a different set of experiments, where its effect on spore capture was also evaluated.

#### Collection time

In order to determine the effect of sampling duration on fungal spore capture efficiency and identify the optimal collection time under optimized airflow rate, five different time intervals were tested: 5, 15, 30, 60, and 90 min. For all the tested sampling durations, 15L/min airflow rate was used.

#### Saturation test

The saturation point of coated (POX and PFO) and non-coated (NC) filters was determined by sampling with an increasing number of plates containing fungal colonies: 1 to 5 plates were used in the sampling chamber. The spores of *A. niger* and *R. glutinis* were aerosolized in the sampling chamber using the optimized flow rate and collection time conditions. Saturation is defined here as the point where adding more source material causes negligible additional mass capture on the filters.

### Optimization of fungal spore quantity

These trials were designed to identify the threshold amount of material capable of producing amplifiable DNA while minimizing sample use for downstream molecular analyses, including DNA extraction and PCR amplification. Previous studies indicate that approximately 50–80 mg of fungal biomass is typically required to achieve reliable DNA extraction and amplification (Conlon et al. 2022; Fryar and Catcheside 2023; Rassinger et al. 2018).

To determine the minimum amount of fungal material necessary to generate consistently amplifiable DNA, a preliminary optimization experiment was performed using all four species: *A. niger*, *Cladosporium* sp., *P. roqueforti*, and *R. glutinis*. Genomic DNA was extracted using a DNeasy® Plant Mini Kit (QIAGEN, Clayton, Australia, Cat. No. 69104). Fungal biomass was collected directly from culture plates, and a range of biomass quantities (2, 4, 6, 8, 12, 14, and 16 mg) was tested. A 50-mg sample was included as a positive control.

### Extraction of spores and viability test

Following air sampling, fungal spores captured on the filters were quantified using two methods: hemocytometry and spore pellet weight as per established procedures (Affan et al. 2026). The filters were transferred into centrifuge tubes containing 1X PBS buffer and vortexed thoroughly to dislodge the spores from the filter surfaces. After vortexing, the filters were carefully removed using sterile forceps.

To assess the viability of the recovered spores, an aliquot of 50 µL from the suspension was spread onto fresh agar plates containing the appropriate growth medium for each fungal species. The plates were incubated under species-specific optimal conditions, as described above, and monitored to confirm spore viability. Plates of *A. niger*, *P. roqueforti*, and *R. glutinis* were incubated for 7 days, while *Cladosporium* sp. plates were incubated for 18 days due to its slower growth. The remaining suspension was centrifuged at 4500 rpm for 5 min to pellet the spores. The obtained spore pellets were dried overnight inside a biosafety cabinet at room temperature, to ensure complete removal of residual moisture and then stored at − 20 °C until subsequent molecular analysis.

### DNA extraction, amplification, and sequencing

Spore pellets recovered from filters were used in DNA extractions. Dried spore pellets were resuspended in the lysis buffer from a Qiagen DNeasy Plant Mini Kit using a micropipette and vortexed at low speed to resuspend all the spores. The spores were crushed using a sterile micropipette for 1 min. Next, genomic DNA was extracted using a Qiagen DNeasy Plant Mini kit according to the manufacturer’s instructions. The final DNA extracts were eluted into 100 mL of elution buffer. A Thermo Scientific NanoDrop One Microvolume UV–Vis Spectrophotometer (Victoria, Australia, Cat NO. 13400518) was used to quantify the amount of DNA in ng/µL.

Primers ITS4/ITS5 (White et al. 1990) were used to amplify the internal transcribed spacer (ITS) regions and the intervening 5.8S rRNA gene. Reaction mixtures contained 1 µL (10 mM each) dNTPs (deoxynucleotide triphosphates), 2.5 µL of each primer (10 mM), 10 µL 5X Phusion HF buffer, 4 µL DNA template, 1.5 µL DMSO (dimethyl sulfoxide), 0.5 µL Phusion High-Fidelity DNA polymerase, and 28 µL sterile milliQ water to a final volume of 50 µL.

PCR amplification was performed in a BIO-RAD (South Granville, NSW, Australia) T100 Thermo Cycler. Cycling conditions for PCR were as follows: initial denaturation at 98 °C for 30 s, 25 cycles of denaturation at 98 °C for 10 s, annealing at 58 °C for 30 s, and extension at 72 °C, followed by final extension at 72 °C for 10 min. PCR amplicons were observed on a 1.5% agarose electrophoresis gel stained with Gel Red (Biotium, Fremont, CA, USA). The resulting amplicons were purified using a Monarch Spin PCR and DNA Cleanup kit (Cat # T1130S, New England Biolabs, Notting Hill, Australia) and prepared according to the instructions of the Australian Genome Research Facility (AGRF, Parkville, Australia) and sent for sequencing in both directions. Raw sequences were assembled, examined, and edited using Unipro UGENE (version 52.1; Unipro, Novosibirsk, Russia). The generated sequences for each sample were used in megablast searches (Zhang et al. 2000) to identify closely related sequences in the National Center for Biotechnology Information (NCBI, Bethesda, USA) GenBank nucleotide database.

#### Sample nomenclature

Plasma treatment of filters was performed in three independent plasma treatment runs, conducted on separate days (runs 1–3) to generate chemical replicates and to account for day-to-day variability in plasma coating conditions. For each run, three plasma-treated filters were prepared and used as technical replicates, designated a–c. Each filter was used in a separate aerosolization experiment, with a freshly prepared agar plate containing a fungal colony, ensuring that each aerosolization experiment was biologically independent.

Sample nomenclature was defined as follows: “P” denotes agar plate–derived samples, and “F” denotes filter-derived samples. Numerical identifiers (1–3) correspond to chemical replicates generated from independent plasma treatment runs on different days, while alphabetical identifiers (a–c) indicate technical replicate filters from the same plasma run. LB denotes the DNA ladder, (–CN) the negative control, and (+ CN) the positive control. Fungal species abbreviations are AN (*A. niger*), CL (*Cladosporium* sp.), PR (*P. roqueforti*), and RG (*R. glutinis*).

### Statistical analysis

Data was analyzed using GraphPad Prism (V10.4.0 (621); Boston, MA, USA). Significance was determined via two-way analysis of variance (ANOVA) with multiple comparisons. Uncorrected Fisher’s least-significant difference (LSD) test was applied to compare the NC (control) and each plasma coating (POX, and PFO) for each tested fungal species. In all cases, *p*-values of ≤ 0.05 were used for significant difference.

## Results

### Optimal airflow rate

**Fig. 1:**
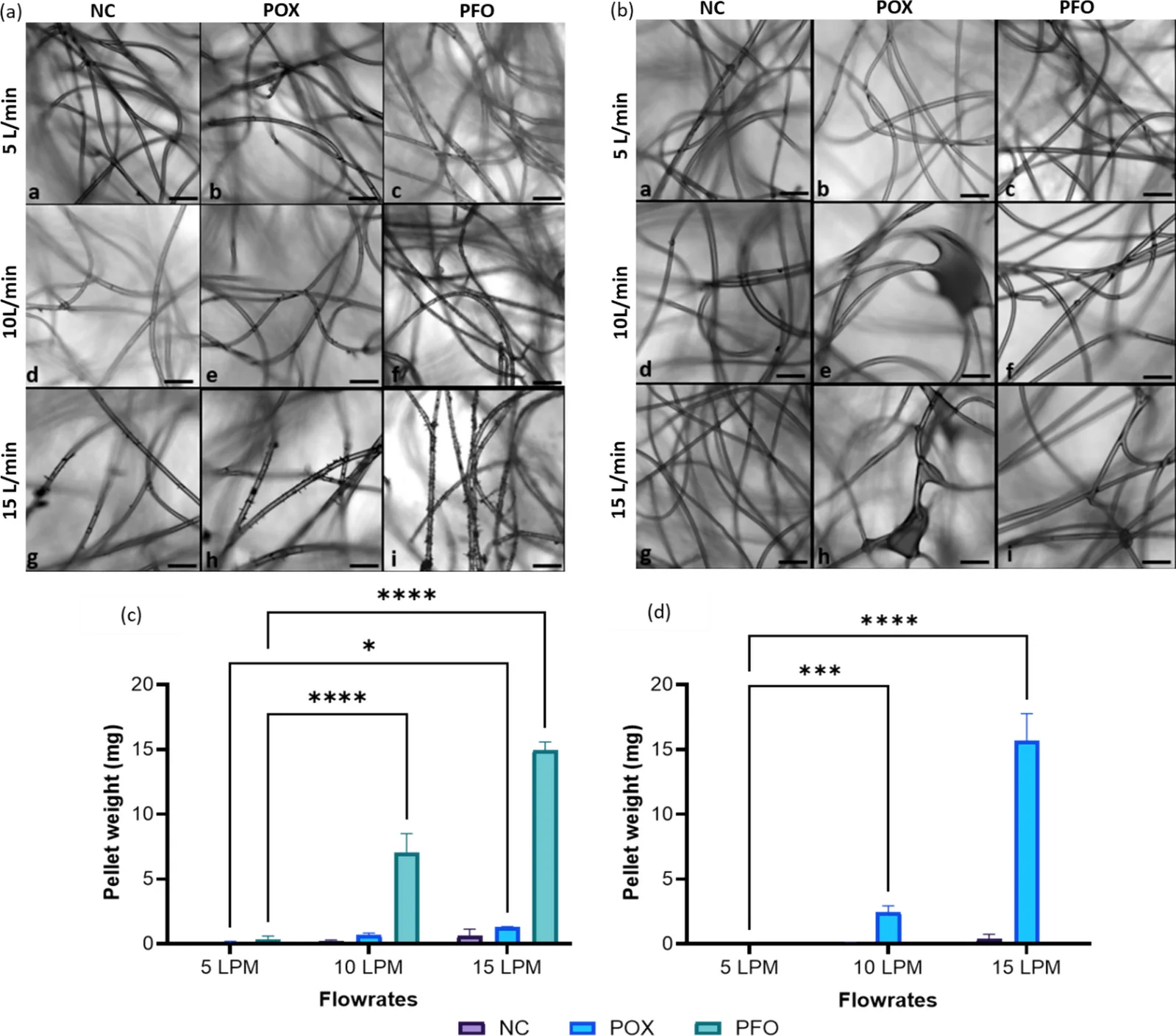
Analysis of different airflow rates (5, 10, 15 L/min) on spore collection (spore mass) over coated (POX, PFO) and non-coated (NC) filters from two fungal species. **a** Microscopic images of filters captured *A. niger* spores. **b** Microscopic images of filters captured *R. glutinis* spores. Images taken =  × 100 magnification. Scale bar = 200 μm. (c–d) Quantification of captured fungal biomass (**c**) *A. niger* and (**d**) *R. glutinis*. Data is expressed as mean ± standard deviation (SD) from three independent replicates (*n* = 3). Error bars represent SD. *p*-value = * ≤ 0.05, ** ≤ 0.01, **** ≤ 0.0001

The influence of airflow rate on fungal spore aerosolization and capture efficiency was investigated using three different flow rates: 5, 10, and 15 L/min. Two representative fungal species, *A. niger* and *R. glutinis*, were tested across three filter types (NC, POX, and PFO) as shown in Fig. 1.

At the lowest flow rate (5 L/min), only minimal spore capture was observed for both species across all filter types. Increasing the flow rate to 10 L/min led to a noticeable improvement in collection efficiency, particularly on the PFO and POX-coated filters (*p* < 0.05).

For both *A. niger* and *R. glutinis*, the most substantial spore capture was observed at 15 L/min, the highest flow rate achievable with the current experimental setup (Fig. 1a–b). Under this condition, the pellet weights increased significantly (*p* < 0.05), indicating enhanced capture of the aerosolized spores by the coated filters. Compared with 5 L/min, the mean collected mass of *A. niger* spores increased to 7.0 mg (standard deviation, SD = 1.4) at 10 L/min and to 14.9 mg (SD = 0.6) at 15 L/min when PFO filters were used (*p* < 0.0001) (Fig. 1c). When comparing the other two filters (NC and POX) at different flow rates, there was no difference for *A. niger* (*p* < 0.05). Similarly, relative to 5 L/min, the mean collected mass increased by 2.4 mg (SD = 0.5) at 10L/min and 15.7 mg (SD = 2.1) at 15 L/min for *R. glutinis*, when POX filters were used for spore sampling (*p* < 0.001 and *p* < 0.0001, respectively), while other comparisons were non-significant (Fig. 1d).

Among the filter types, PFO-coated filters exhibited the highest spore collection of 14.9 mg (SD = 0.6) for *A. niger* (Fig. 1c), while POX-coated filters captured 15.7 mg (SD = 2.1) of *R. glutinis* spores (Fig. 1d). In contrast, the non-coated (NC) filters consistently exhibited the lowest capture efficiency, resulting in minimal pellet mass under all conditions investigated, with highest values of 0.6 mg (SD = 0.5) for *A. niger* and 0.4 mg (SD = 0.3) for *R. glutinis* at 15 L/min.

### Optimal collection time

**Fig. 2:**
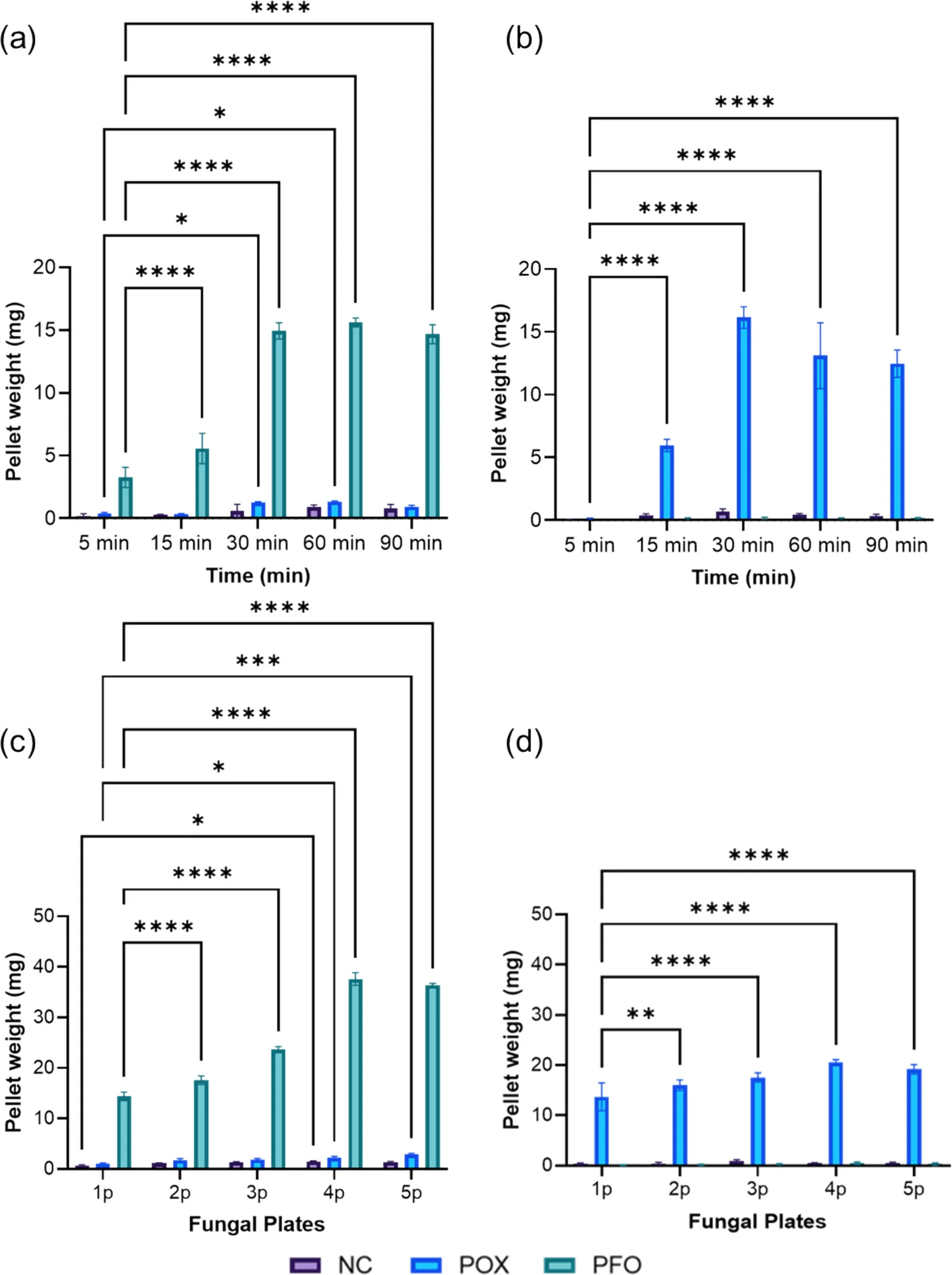
Comparative analysis of sampling duration effects and saturation behavior of coated and non-coated filters. **a**–**b** Effect of sampling duration on spore mass recovery. **c**–**d** Saturation point of filters at variable fungal source material. **a**, **c** *A. niger*. **b**, **d** *R. glutinis*. Data is presented as mean ± standard deviation (SD) from three independent replicates (*n* = 3). Error bars represent SD; for some results, error bars are small and partially obscured by the data markers due to low variability

The effect of sampling duration on spore capture efficiency was evaluated at the previously optimized airflow rate of 15 L/min using *A. nige*r and *R. glutinis* (Fig. 2a, b). Different collection times were tested (5, 15, 30, 60, and 90 min) using NC, POX, and PFO filters to determine whether prolonged sampling increased the amount of collected spore biomass and evaluated microscopically (Supplementary Fig. S1).

For *A. niger* (Fig. 2a), the PFO filter showed a marked increase in captured biomass between 5 and 30 min, after which the pellet weight reached a steady plateau at 15 mg. Compared to the 5-min sampling duration, PFO-coated filters showed a significant increase in retained mass at all subsequent time points (15–90 min; *p* < 0.0001). In contrast, both NC and POX filters exhibited much lower and relatively stable *A. niger* spore pellet weights throughout the tested time ranges, showing minimal variation as duration increased. No differences were observed across sampling durations for NC filters (*p* > 0.05). For POX-coated filters, increases were observed only at 30 and 60 min relative to 5 min (*p* < 0.05).

A similar trend was observed for *R. glutinis* with POX filters yielding higher pellet weights than NC and PFO filters, followed by stabilization after 30 min across all time points (Fig. 2b). Specifically, compared to 5-min sampling duration, the fungal mass captured on POX-coated filters increased at all subsequent time points for *R. glutinis* (15–90 min; *p* < 0.0001). No differences were observed across sampling durations for NC and PFO filters (*p* > 0.05).

### Saturation test

The spore saturation point of coated and non-coated filters was evaluated by varying the number of fungal source plates used during sampling at the previously optimized airflow rate of 15 L/min and a sampling duration of 30 min. An increasing number of agar plates containing *A. niger* and *R. glutinis* colonies (1–5 plates) were aerosolized within the sampling chamber and observed microscopically after sampling (Supplementary Fig. S2).

The amount of *A. niger* spores captured by PFO filters was markedly more than those captured by either NC or POX (*p* < 0.0001). With 5 plates, PFO-coated filters captured a mean of 36.4 mg (SD = 0.3) of spores compared with NC filters capturing 1.33 mg (SD = 0.2) and POX filters 2.9 mg (SD = 0.2). For *A. niger* the amount of fungal material captured by PFO filters increased with the number of fungal plates from 1 to 4, reaching a maximum capture of 37.6 mg (SD = 1.2) (*p* < 0.0001) (Fig. 2c). Beyond this point, any additional fungal plates did not increase the amount of spores captured, indicating that the PFO filters had reached their saturation capacity (*p* > 0.05). In contrast, both NC and POX filters showed minimal changes across all plate numbers, suggesting low *A. niger* spore retention efficiency and the absence of saturation within the tested range (*p* > 0.05). Using NC filters, there was only a slight difference between 1 and 4 plates (*p* = 0.047). For POX filters, increases were observed at higher plate numbers, specifically between 1 vs. 4 plates (*p* = 0.011) and 1 vs. 5 plates (*p* = 0.0001).

A different trend was observed for *R. glutinis* (Fig. 2d), where the POX filters demonstrated the highest spore capture, gradually increasing with higher source concentrations before plateauing after 4 plates. The NC and PFO filters consistently captured negligible amounts of *R. glutinis* fungal material, and these values were not significant. The POX filters thus exhibited superior performance in terms of spore capture efficiency and defined saturation behavior for *R. glutinis* compared to the other filter types (*p* < 0.0001) (Supplementary Fig. S3). POX filters exhibited significant increases relative to 1 plate at all higher plate numbers (1 vs. 2, *p* = 0.0029; 1 vs. 3–5, *p* < 0.0001). These results indicate a strong and progressive increase in spore capture for POX filters.

### Viability of spores captured on filters

This experiment was conducted with *A. niger*, *Cladosporium* sp., *P. roqueforti*, and *R. glutinis* to determine whether the plasma polymer coatings applied to the filters interfered with the viability and subsequent colony formation of the captured fungal spores.

**Fig. 3:**
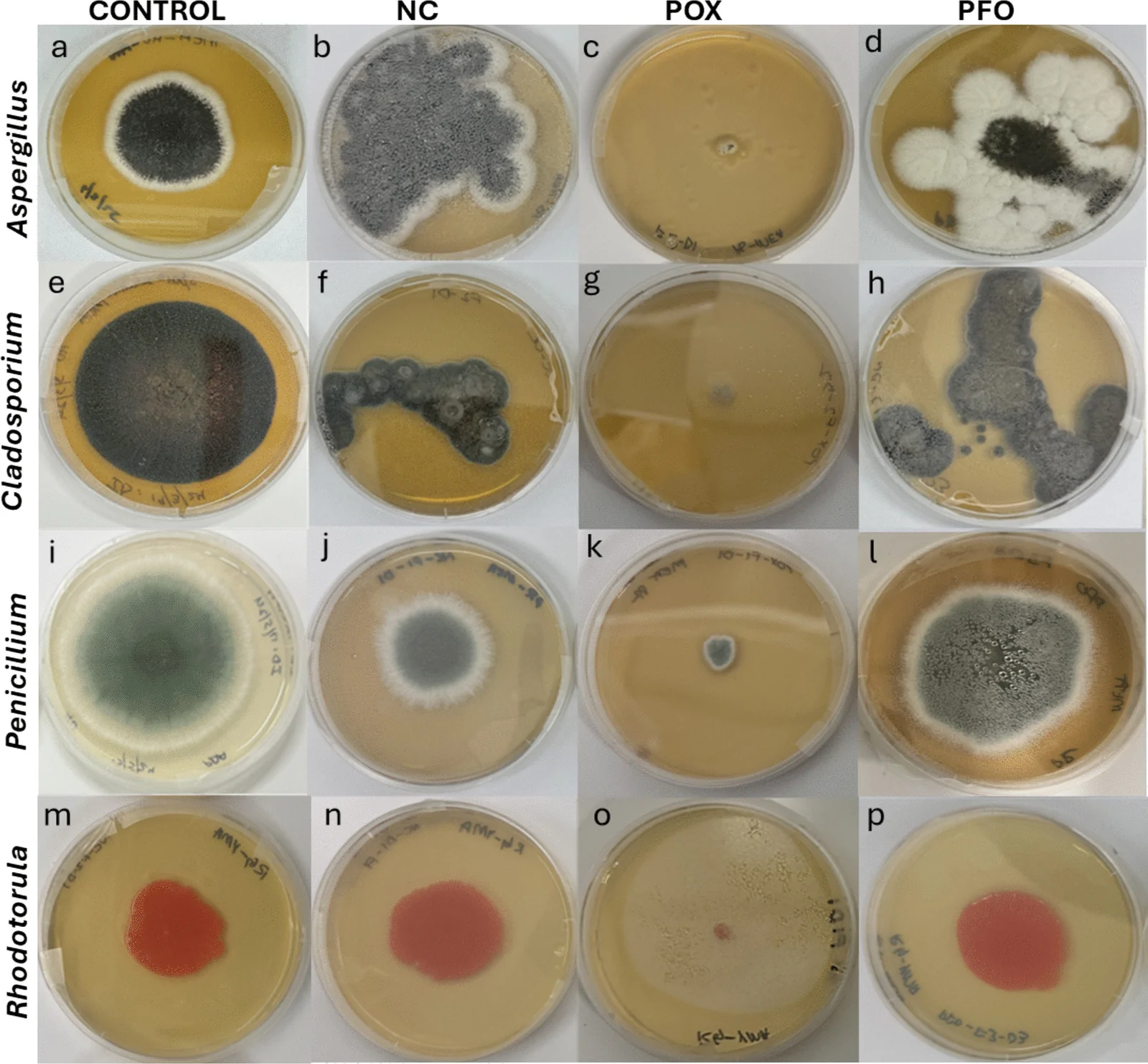
Spore viability test by using the spore solution extracted from coated (POX, PFO) and NC filters comparing against control plates for each strain. Columns showing: CONTROL, NC, POX, and PFO filters respectively. Rows showing different fungal species colonies growth on agar plates **a**–**d** *A. niger*, **e**–**h** *Cladosporium* sp., **i**–**l** *P. roqueforti*, and **m**–**p** *R. glutinis*. Images were captured after 7 days (*A. niger*, *P. roqueforti*, *R. glutinis*) and 18 days (*Cladosporium* sp.) of incubation

All four species exhibited clear colony formation after elution from the NC and PFO filters, confirming that the spores remained viable following the extraction process. These were compared against the control fungal colonies grown directly from stock cultures to assess any differences in growth morphology, colony diameter, pigmentation, and overall viability (Fig. 3). The fact that the spores extracted from NC and PFO-coated filters successfully regrew on the agar plate.

In contrast, the spores extracted from the POX-coated filters form smaller and less numerous colonies after the incubation period for all tested fungal species (Fig. 3). Quantitative analysis from our previous work demonstrated that, for all filamentous fungi (*Aspergillus*, *Cladosporium*, *Penicillium*), both POX and NC filters captured less spores as compared to PFO filters, indicating that differences in initial spore load being put onto the plates may partly contribute to the observed outcomes. However, this factor alone does not fully explain the reduced colony development. Notably because, for *Rhodotorula* (yeast), POX filters exhibited the highest spore capture efficiency, yet colony formation remained limited after incubation. Reassessment of the extraction procedure confirmed that the washing and agitation steps did not compose spore integrity, as evidenced by successful regrowth from NC and PFO filters. To further evaluate whether the filter coatings influence downstream analytical performance beyond culturability, DNA extraction and PCR analyses were subsequently conducted to assess their compatibility with molecular identification methods.

### Optimization of fungal spore quantity for molecular analysis

The quantified DNA concentrations derived from varying quantities of fungal biomass for each of the four species strains are shown in Supplementary Fig. S4. To evaluate what spore quantity was adequate for DNA extraction and PCR, controlled extractions were performed using increasing amounts of fungal biomass (2–16 mg), with a 50-mg sample included as a positive control.

Nucleic acid concentration remains essentially negligible at low sample weights, with the values close to zero between 2- and 12-mg sample weight for all four species accompanied by relatively large error bars that indicate considerable variability among replicates. DNA concentration increased sharply once the captured fungal biomass reached ~ 14 mg, with a clear threshold effect, where concentration jumped to roughly 18–20 ng/µL. Increasing the sample weight further, up to 50 mg, did not result in a further increase of the nucleic acid concentration and the variability observed at higher biomass levels suggests that DNA recovery becomes limited by extraction efficiency rather than by the amount of fungal material.

### PCR and gel electrophoresis

**Fig. 4:**
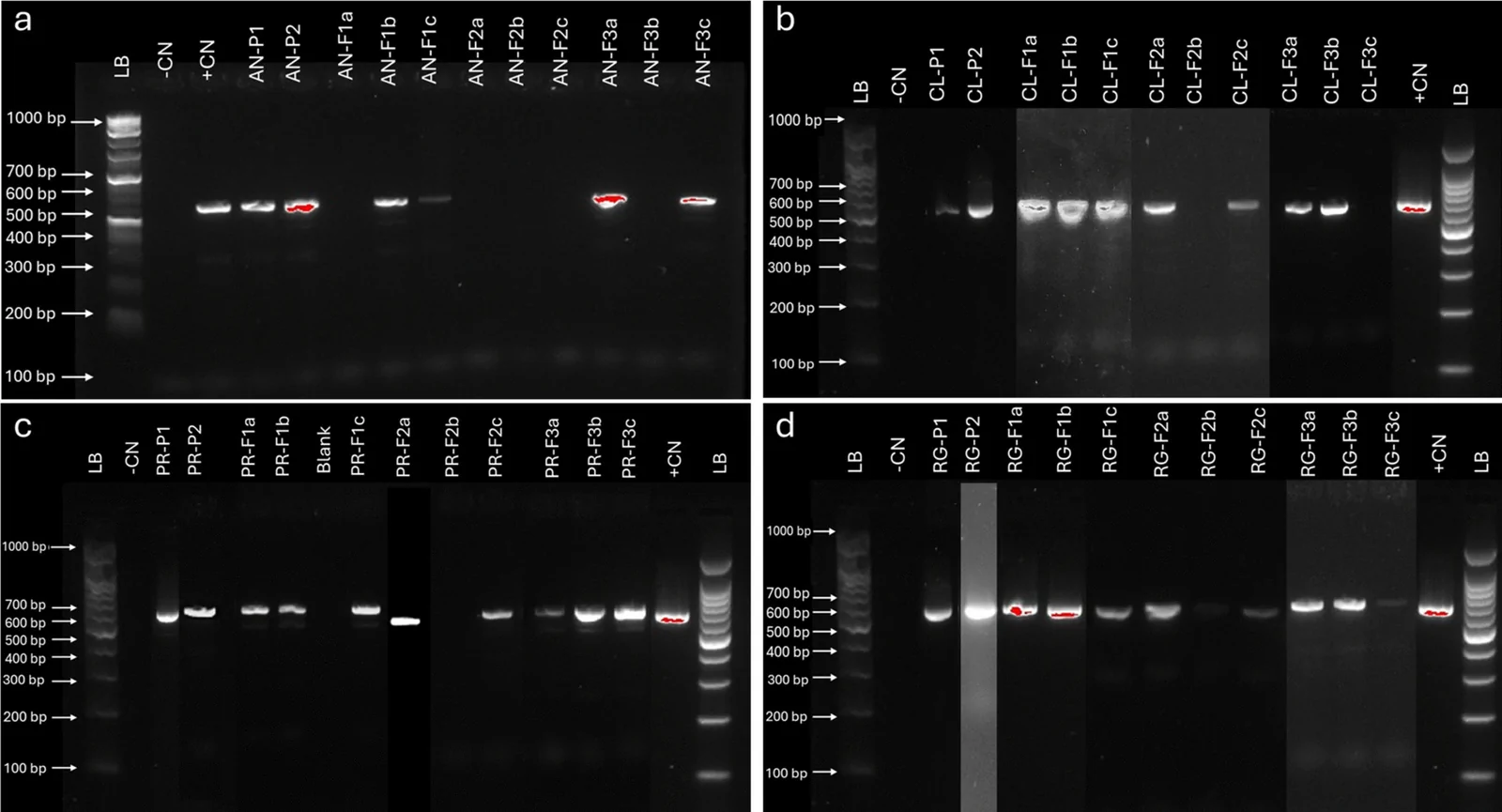
PCR-based detection different fungal species using the primers such as ITS4 and ITS5. **a** *A. niger* (AN). **b** *Cladosporium* sp. (CL). **c** *P. roqueforti* (PR). **d** *R. glutinis* (RG). Lanes representative: LB, DNA ladder; P, samples obtained from plates; 1–3, chemical replicates; a–c, technical replicates; F, samples obtained from filters; -CN, negative control; + CN, positive control

PCR amplification of fungal DNA was achieved, with agarose gel electrophoresis revealing distinct and sharp bands within the expected size range (~ 550–600 bp) for the ITS region. Bands corresponding to *A. niger* (AN), *Cladosporium* sp. (CL), *P. roqueforti* (PR), and *R. glutinis* (RG) were observed in the tested samples, although amplification was not detected in all individual replicates, as shown in Fig. 4.

Amplicon sizes were consistent with those obtained from the respective reference isolates used as positive controls (+ CN), indicating successful amplification. In addition, PCR products derived from spores collected on polymer-coated filters exhibited band sizes and intensities comparable to those generated from reference culture DNA, demonstrating that neither the sampling process nor the DNA extraction compromised nucleic acid integrity or species-level identification. No amplification was observed in the negative controls, confirming the absence of contamination. *A. niger*, *Cladosporium* sp., and *P. roqueforti* were successfully recovered from PFO-coated filters, while *R. glutinis* was recovered from POX-coated filters. Comparable band intensity and size were obtained across both coating treatments (POX and PFO) for successfully amplified samples.

For *A. niger* (Fig. 4a), PCR amplification was inconsistently detected across PFO filter samples, with several replicates yielding no visible amplicon despite pellet masses within the 15–17 mg range (AN-F1a, AN-F2a–c, AN-F3b). Similarly, *Cladosporium* sp. recovered from PFO filters showed non-detection in individual replicates at both lower (12.3 mg; CL-F2b) and higher (14.7 mg; CL-F3c) pellet masses (Fig. 4b).

For *P. roqueforti*, only one out of nine replicates (PR-F2b) did not produce a detectable band despite a pellet mass of 14.6 mg (Fig. 4c). A comparable pattern was observed for *R. glutinis* collected on POX filters, where the RG-F2b replicate (15.4 mg) showed a very faint band at the expected ITS amplicon size, suggesting low-level amplification (Fig. 4d).

Across all species, the proportion of non-amplifying replicates varied, with approximately 55% of replicates failing to amplify for *A. niger*, 22% for *Cladosporium* sp., and 11% for *P. roqueforti*. Notably, *R. glutinis* appeared more consistently detectable than the other species. Amplification appeared more consistent for samples collected on POX-coated filters. Notably, for PFO, the second plasma treatment run accounted for 5 of the 9 samples that showed non-amplification. Overall, these results show no consistent correlation between recovered pellet mass and successful ITS amplification.

### Sequencing results

The ITS region of fungal isolates obtained from both plates and filters was successfully amplified and sequenced. The ITS sequences were analyzed using the megablast searches against the NCBI GenBank reference sequences derived from taxonomically validated type material to confirm species-level identity, as shown in Table 1.

**Table 1:** List of fungal species analyzed in this study, with GenBank accession numbers, percentage identity, and collection material (filter or plate)

Sample ID	Material collected	Species culture name	Closest GenBank accession	% identity	Species name in GenBank	Remarks	Ex-type cultures in GenBank
CL-P1	Plate	*Cladosporium* sp.	NR_119608.1	100%	*C. fusiforme*	Closely related species	Type material
CL-P2	Plate	*Cladosporium* sp.	NR_119608.1	100%	*C. fusiforme*	Closely related species	Type material
RG-P1	Plate	*R. glutinis*	NR_073294.1	99.49%	*R. glutinis*	Species confirmed	Type material
PR-P1	Plate	*P. roqueforti*	NR_103621.1	99.65%	*P. roqueforti*	Species confirmed	Type material
AN-P1	Plate	*A. niger*	AY373852.1	100%	*A. niger*	Species confirmed	Type material
AN-P2	Plate	*A. niger*	U65306.1	100%	*A. niger*	Species confirmed	Type material
AN-F1b	Filter	*A. niger*	AY373852.1	100%	*A. niger*	Species confirmed	Type material
AN-F1c	Filter	*A. niger*	NR_111348.1	100%	*A. niger*	Species confirmed	Type material
AN-F3a	Filter	*A. niger*	NR_111348.1	100%	*A. niger*	Species confirmed	Type material
AN-F3c	Filter	*A. niger*	NR_111348.1	100%	*A. niger*	Species confirmed	Type material
CL-F1c	Filter	*Cladosporium* sp.	LT907958.1	99.60%	*C. michoacanense*	Closely related species	Type material
CL-F2a	Filter	*Cladosporium* sp.	LT907958.1	99.60%	*C. michoacanense*	Closely related species	Type material
CL-F3b	Filter	*Cladosporium* sp.	LT907958.1	99.60%	*C. michoacanense*	Closely related species	Type material
PR-F1c	Filter	*P. roqueforti*	NR_103621.1	100%	*P. roqueforti*	Species confirmed	Type material
PR-F2a	Filter	*P. roqueforti*	Noise in sequences				
PR-F3c	Filter	*P. roqueforti*	MH855127.1	100%	*P. roqueforti*	Species confirmed	Type material
RG-F1b	Filter	*R. glutinis*	NR_073294.1	99.49%	*R. glutinis*	Species confirmed	Type material
RG-F1c	Filter	*R. glutinis*	NR_073294.1	99.49%	*R. glutinis*	Species confirmed	Type material
RG-F2a	Filter	*R. glutinis*	NR_073294.1	99.50%	*R. glutinis*	Species confirmed	Type material
RG-F3a	Filter	*R. glutinis*	NR_073294.1	99.49%	*R. glutinis*	Species confirmed	Type material
RG-F3b	Filter	*R. glutinis*	NR_073294.1	99.49%	*R. glutinis*	Species confirmed	Type material

BLAST analysis confirmed that all sequences corresponded to the expected fungal species used in the experiments, with sequence similarity values ranging between 99.4 and 100% when compared against type material reference sequences. *A. niger*, *P. roqueforti*, and *R. glutinis* showed 99–100% identity with their respective GenBank accessions across both plate and filter samples, confirming species consistency and indicating that the plasma coatings did not interfere with DNA quality, amplification, or sequencing fidelity. All *A. niger* isolates matched the reference sequences (AY373852.1 and NR_111348.1) with 100% identity, demonstrating full reproducibility between experimental replicates.

Similarly, *P. roqueforti* isolates yielded 99.6–100% sequence similarity to the reference NR_103621.1, while *R. glutinis* sequences consistently showed 99.49–99.5% identity to NR_073294.1, confirming reliable species-level identification. One sample (PR-F2a) produced noisy chromatograms with unreadable sequence data, while all other samples yielded clean chromatograms with sharp peaks.

For *Cladosporium*, however, plate-derived samples (CL-P1 and CL-P2) matched *C. fusiforme* with 100% sequence identity, while filter-derived isolates (CL-F1c, CL-F2a, and CL-F3b) showed 99.6% identity to *C. michoacanense*.

Importantly, the sequencing was successfully performed using DNA extracted from approximately 14–16 mg of fungal material captured on coated filters, demonstrating that this biomass amount was sufficient for downstream molecular testing, including PCR amplification and ITS sequencing. Captured fungal material also retained biological functionality, as spores collected on PFO filters were successfully regrown into viable colonies.

## Discussion

This study investigates that plasma polymer surface modifications substantially improve fungal spore capture compared with non-coated (NC) PET filters. Across the airflow optimization, sampling duration, and saturation experiments, coated filters consistently recovered greater quantities of fungal biomass as compared to NC filters.

The results demonstrated that airflow rate is a critical factor of fungal spore aerosolization and capture efficiency. The lower recovery was observed at 5 L/min tested airflow; this low recovery was attributed to insufficient airflow strength to effectively aerosolize and transport the fungal spores towards the filter surface. This suggests that spore detachment and suspension are limited under weak dynamic conditions (Li 2022). As airflow increased to 10 L/min, improved spore recovery was observed, particularly for coated filters, indicating enhanced aerosolized spores generation and transport. However, the total spore recovery at this intermediate flow rate remained moderate compared to the highest flowrate tested, which suggest that spore-filter interactions and transport mechanisms are not fully optimized (Li and Fu 2020). The significantly higher fungal spore capture was observed at 15 L/min and highlights the importance of increased airflow rate in promoting enhanced aerosolization, transport, and deposition of fungal spores. Higher airflow enhances inertial impaction and interception, key mechanisms governing deposition of fungal spores (typically 1–30 µm). This agrees with bioaerosol sampling studies reporting higher recovery using active high-flow systems that process larger air volumes (Haas et al. 2017; Li et al. 2021).

Although the airflow rates investigated (5–15 L/min) enabled reliable fungal spore collection under laboratory conditions, collection efficiency under passive natural environmental conditions may differ due to variations in ambient airflow, turbulence, and spore (or other particles) transport processes. Furthermore, while extremely low airflow rates may reduce particle delivery to the collector and thus decrease capture efficiency, the present study was not designed to determine the minimum airflow threshold required for fungal spore collection. Future studies should evaluate sampler performance across a broader range of airflow conditions, including real-world environmental conditions.

In addition to airflow, the results highlight the critical role of plasma coatings in determining capture efficiency. The superior performance of PFO filters for *A. niger* and POX filters for *R. glutinis* suggest that spore-filter surface interactions are governed by physicochemical properties. The consistently low performance of NC filters confirms that surface modifications enhanced fungal spore adhesion and retention. Overall comparative results from these trials identified 15 L/min as the optimal operating condition for all other experiments in this study. However, it cannot be excluded that flow rates higher than 15 L/min could yield further increases in capture efficiency, as a clear performance plateau was not reached within the range investigated.

Spore recovery plateaued within the first 30 min of sampling, suggesting that the majority of aerosolized spores were collected in this sampling period. The contrasting performance between filters indicates differences in capture efficiency, with retention of *A. niger* on PFO and *R. glutinis* on POX, while NC and POX (for *A. niger*) or NC and PFO (for *R. glutinis*) appeared to reach saturation more quickly likely due to weaker spore-surface interactions. The plateau observed at longer sampling durations indicates that spore collection approached a steady state, with no further increase in recovered mass beyond 30 min. This plateau in spore recovery likely occurred because loosely attached spores aerosolized early in the experiment, while the remaining spores stay attached to the source plates under the applied airflow conditions.

The study further demonstrates clear differences in spore capture efficiency and saturation behavior between coated and non-coated filters, as well as between fungal species with distinct dispersal strategies. Both fungal spore characteristics and filter surface chemistry plays a critical role in recovery. The plateau in filter-collected biomass observed for *R. glutinis* despite increasing the numbers of source plates indicates that airborne recovery was constrained by aerosolization efficiency rather than by the amount of available fungal biomass, reflecting intrinsic differences in fungal dispersal strategies. As a surface-associated yeast producing large, wet cells embedded within extracellular matrices, *R. glutinis* showed minimal airborne release and rapidly reached a recovery plateau, consistent with the limited contribution of yeasts to airborne fungal communities in indoor environments (do Nascimento et al. 2023).

In contrast, *A. niger* exhibited an increase in recovered mass with increasing plate number (up to 4 plates), consistent with its ability to release abundant airborne spores. Filamentous species like *Aspergillus* produce vast numbers of small (≈2–4 µm), dry, hydrophobic spores that are specifically adapted for wind-mediated dispersal and detach readily under low airflow shear forces, contributing significantly to airborne fungal populations in both indoor and outdoor environments (Kwon-Chung and Sugui 2013). These hydrophobic spores are easily entrained by airflow, and their small aerodynamic diameters further enhance their capacity to remain suspended and be collected on filters (Cai et al. 2020). However, a plateau was also observed for *A. niger* at 4 plates. The mechanism underlying this response is uncertain. While aerosolization limitations may contribute, an alternative explanation is saturation of the filter’s capture capacity at higher spore loads. Thus, capture plateaus may result from different limiting processes depending on fungal species and spore characteristics.

Overall, these results are in agreement with observations made in the previous airflow rate and collection time optimization experiments, where coated filters showed greater spore capture efficiency as compared to NC. These plasma polymer coatings substantially enhance the spore capture capacity of the filters. The saturation of filters observed at 4–5 fungal plates defines the operational limit of these filters under the current sampling conditions, providing a basis for optimizing future airborne fungal spore sampling and DNA extraction protocols.

The findings also highlight the importance of surface chemistry which plays a critical role in influencing apparent viability and recovery of fungal spores following capture and extraction. In particular, the reduced colony size and number observed for spores extracted from POX filters across all tested species indicate that the POX surface chemistry may influence the apparent viability of the captured spores. However, this reduction effect cannot be unequivocally attributed to reduced viability alone. Alternative explanations such as lower germination efficiency of the recovered spores or increased aggregation may also result in fewer colony-forming units despite similar spore numbers. In addition, interactions with the coating material during capture or extraction may also affect spore dispersion prior to plating. Therefore, although the results point towards a potential inhibitory effect of the POX coating, targeted viability and germination assays would be required for confirmation.

The observed reduced colony size and number observed for spores extracted from POX-coated filters are consistent with previous reports describing the intrinsic antimicrobial activity of plasma-deposited poly(oxazoline) coatings. More broadly, poly(2-oxazoline)s-based materials and related oxazoline-derived polymers have been widely investigated as antimicrobial and antibiofouling surface coatings, with demonstrated activity against a broad range of microorganisms (Cavallaro et al. 2016; He et al. 2016; Oleszko-Torbus et al. 2025). Previous work indicates that oxazoline-based coatings negatively affect microbial viability through surface-contact mechanisms, including disruption of membrane integrity, changes in surface hydration, and inhibition of adhesion, leading to reduced microbial proliferation even in the absence of complete cell lysis (Alkarri et al. 2024; Concilio et al. 2023). Although much of the existing literature focuses on antibacterial performance, antifungal effects have also been described for antimicrobial polymer coatings, where fungal spores exhibit delayed germination, reduced hyphal development, and diminished colony formation following surface contact (Haktaniyan and Bradley 2022). In this context, the smaller and less numerous colonies recovered from POX-coated filters suggest a sub-lethal but biologically relevant impact on fungal spores, likely arising from chemical interactions between the POX surface and the spore cell envelope or interference with the extraction process. Similar surface-contact-dependent effects have been reported for antimicrobial polymer coatings, where viability is not entirely eliminated but growth and metabolic activity are significantly suppressed following exposure (Alkarri et al. 2024; Babutan et al. 2021).

In contrast, the successful recovery and regrowth of fungal spores extracted from PFO filters indicates that the surface chemistry of the filters did not negatively influence fungal viability. This observation is particularly important because the recovery of viable airborne fungi is known to depend strongly on sampling conditions, airflow exposure, sampling duration, and filter material properties. Conventional dry filters, including cellulose ester, glass fiber, and polycarbonate (PC), have frequently been associated with reduced fungal culturability because prolonged sampling and continuous air flow can cause desiccation (Eduarda and Heederik 1998; Nasiri et al. 2021; Nevalainen et al. 1992). Under these conditions, fungal spores may lose water, experience structural damage, or undergo physiological stress that reduces their ability to germinate after collection. These effects have been reported for environmentally relevant fungi such as *Cladosporium* sp., where prolonged exposure to dry, high-velocity air streams leads to reduced spore germination and colony formation after collection (Tang et al. 2015).

Within the broader context, filter surface chemistry may influence the extent to which spores remain viable after capture in two ways. First, improved capture efficiency could mean that shorter sampling times can be used. Second, hydrophobic interactions between fungal spores and the filter surface can affect spore adhesion, retention, and potentially the degree of physical stress experienced during sampling and recovery (Fernandes et al. 2019; Maus et al. 2001). In the present study, while the PFO coating improved fungal spore capture efficiency, it is not expected to completely prevent viability losses associated with prolonged dry air sampling, though it could reduce sampling times. Rather, the key finding from this study is that the PFO surface did not appear to introduce additional inhibitory or antimicrobial effects that would compromise fungal recovery.

It is also worth noting that filter surface treatments such as UV, iodine, and antimicrobial coatings are sometimes intentionally applied to filtration materials with the aim to suppress microbial survival, inhibit fungal growth, and reduce biofilm formation (Huang et al. 2010; Jeong and Kim 2021; Nakpan et al. 2019; Näsman et al. 1999). In contrast, the PFO coatings used in this study were not intended to exert antimicrobial activity, but rather to enhance the fungal spore capture while still allowing viable spores to be recovered, supporting its base suitability for applications where both spore collection and subsequent culture based or molecular analysis are required. This distinction is important because it highlights that unlike antimicrobial filtration approaches that prioritize microbial inactivation, the PFO-coated filters were able to improve capture efficiency while maintaining biological viability.

Finally, reassessing the viability of fungal spores after extraction was an important step to confirm that the washing and agitation processes did not compromise spore integrity. Importantly, the successful recovery and regrowth of fungal spores captured on PFO filters demonstrated that this plasma polymer coating is compatible with culture-based analysis for colony identification and molecular workflows. Following this confirmation of spore viability, DNA extraction and PCR analyses were performed to further assess whether the filter coatings interfered with molecular-level processes such as DNA amplification and identification. These subsequent experiments aimed to provide insight into the compatibility of each coating with downstream molecular methods.

Furthermore, the optimization tests were designed to determine whether the amount of fungal biomass recovered during the air sampling experiments was sufficient for downstream molecular analysis. The results indicate that consistent nucleic acid recovery is only achieved once biomass approaches ~ 14 mg. Below this threshold, insufficient biological material results in very low DNA yield and high variability across replaces indicating inefficient nucleic acid recovery. The upper limit of 16 mg was selected as it reflects the maximum biomass that could be consistently recovered under the current sampling conditions, particularly for *R. glutinis*, even when multiple source plates were used. The absence of further increase in DNA concentration at higher biomass levels suggests that once sufficient material is available, DNA extraction becomes constrained by methodological limitations rather than biomass availability. This indicates that improving extraction efficiency may be more critical than increasing biomass beyond this threshold. Propagation through spore germination before DNA extraction could increase biomass when less than approximately 14 mg is recovered. However, this additional step may selectively enrich spores that are viable and readily culturable under optimized growth conditions. Direct extraction was therefore used in the present study to evaluate the material recovered directly from the filters without introducing an additional culture-dependent selection step. Nevertheless, propagation may be considered in future applications when the collected biomass is below the threshold required for reliable direct DNA extraction and when obtaining sufficient DNA is more important than preserving the original composition of the captured material.

PCR results confirm that the surface monomer coatings applied to the filters did not interfere with DNA extraction, PCR amplification, or subsequent visualization. These species identities were subsequently confirmed by ITS sequencing (Table 1). Collectively, these results support the compatibility of the coated filters with molecular analyses. Despite overall successful amplification, the results indicate that PCR inconsistency did not show a simple correlation with pellet mass: several samples with ≥ 15 mg biomass did not amplify, whereas some samples with lower biomass were successfully detected. This suggests that additional factors also influence application success. The agreement in amplicon size and integrity between suggesting that filter surface chemistry may influence downstream molecular recovery, potentially through improved wetting and nucleic acid release during extraction.

Species-specific differences likely contributed to this variability. *R. glutinis* showed more consistent amplification, which relate to the fact that the yeast species have softer spore walls, which are more amenable to lysis and DNA extraction (Vishnuvardhan and Joseph 1995). In contrast, the thicker, melanized walls of *Aspergillus* and *Penicillium* spores may reduce DNA recovery efficiency (Halstensen 2008; Yuan et al. 2023). This suggests that factors such as spore physiology, extraction efficiency, and PCR inhibition (Karakousis et al. 2006), rather than pellet mass alone, drive variability. The observed non-amplifying samples within a single PFO plasma treatment run suggest that run-to-run variability in the plasma treatment may further influence molecular recovery. This level of stochastic PCR dropout is consistent with low-biomass environmental DNA studies (Bender et al. 2018; Erb-Downward et al. 2020; Kelly et al. 2019), where subsampling effects, DNA degradation, and uneven spore lysis frequently produce replicate-to-replicate variability. Overall, these results demonstrate that while plasma-polymerized filters successfully capture fungal biomass, molecular detection becomes probabilistic near the lower DNA recovery limit, and replicated sampling is therefore essential for reliable detection of airborne fungi.

Sequencing results confirm that the developed sampling and extraction approach enable reliable molecular identification of captured fungi. High sequence similarity (99.4–100%) across all tested species demonstrates that plasma-polymerized filters do not interfere with DNA quality, amplification, or sequencing fidelity.

The successful sequencing of DNA derived from approximately 14–16 mg of fungal biomass further supports the threshold identified during optimization, indicating that this quantity is sufficient for downstream molecular analysis. This finding confirms that even low quantities of fungal biomass collected on plasma-coated filters can provide molecular identification results, though the variability observed in amplification success rates across species suggests that optimization of DNA protocols and the use of alternative DNA polymerases could further improve amplification consistency and sequencing outcomes. However, the failure of one sample (PR-F2a) suggests that low template concentration or residual inhibitors may still affect sequencing outcomes, highlighting the need for continued optimization of DNA extraction protocols or polymerase selection.

The discrepancy observed in *Cladosporium* species identity reflects a known limitation of ITS region which shows very limited interspecific divergence within *Cladosporium*, and this has been widely reported for this genus (Bensch et al. 2010, 2015; Rosado et al. 2019; Schubert et al. 2007). Although plate-derived samples matched *C. fusiforme* (100% ITS identity) and filter-derived isolates matched *C. michoacanense* (99.6%), these species belong to the *C. cladosporioides* species complex, in which ITS sequences from type strains differ by < 0.5%, which is below the threshold generally accepted for reliable species-level discrimination in fungi. Therefore, all isolates are considered to represent the same *Cladosporium* taxon at the species-complex level, and the differing database assignments reflect the limited taxonomic resolution of the ITS region rather than true biological differences.

Beyond molecular identification, the ability to culture viable fungi from PFO-coated filters used in this study represents a significant functional advantage. The retained viability of captured spores enables downstream taxonomic verification, strain preservation, and further physiological or pathogenicity investigations. This functionality is particularly valuable in bioaerosol research, where discrepancies between molecular detection and viable counts are common. Overall, these findings demonstrate that plasma-polymerized filters are suitable for integrated fungal bioaerosol monitoring, supporting both molecular and culture-based analyses, although further optimization may improve consistency in sequencing outcomes.

## Conclusion

In conclusion, this study establishes a controlled laboratory-based air-sampling workflow to evaluate plasma-polymerized filters for fungal spore collection and compatibility with molecular analyses. Plasma polymerization using POX and PFO monomers produced functionalized filter surfaces that enhanced fungal spore capture efficiency compared to non-coated filters. Among the coatings, PFO consistently demonstrated superior performance in spore capture for all filamentous species tested, likely due to its favorable surface properties facilitating spore adhesion and retention.

Optimization of sampling parameters revealed that an airflow rate of 15 L/min and a sampling duration of 30 min provided the most effective conditions for fungal spore aerosolization and capture across both filamentous (*A. niger*) and yeast-like (*R. glutinis*) species in this experimental setup. Prolonged sampling beyond 30 min did not significantly increase spore biomass recovery, indicating that a steady-state equilibrium was achieved within this period.

The viability tests confirmed that spores recovered from PFO-coated and non-coated (NC) filters retained their ability to regrow on agar media, whereas POX-coated filters exhibited inhibitory effects on colony formation, suggesting a potential antimicrobial interaction with the POX surface chemistry. In contrast, DNA extracted from POX filter samples consistently yielded clear PCR amplification and sequencing results. While amplification from PFO filter samples was also observed but was not consistent across all replicates, suggesting some variability in molecular recovery. Overall, these findings indicate that coating type may influence downstream molecular performance, with POX-coated filters showing comparatively consistent amplification under the conditions tested.

The DNA extraction experiments further demonstrated that approximately 14–16 mg of fungal biomass was sufficient to yield amplifiable and high-quality DNA suitable for ITS-based molecular identification. DNA extracted from spores collected on coated filters was of comparable quality to that obtained from culture plates, indicating that the coatings did not substantially compromise nucleic acid integrity although variability in amplification efficiency was observed between coating types and replicates. Sequencing results validated the accurate species identification of *A. niger*, *Cladosporium* sp., *P. roqueforti*, and *R. glutinis*, with 99–100% similarity to GenBank reference sequences, demonstrating the reliability of the developed sampling and molecular workflow.

Overall, this study establishes plasma-polymerized filters, particularly those coated with PFO as efficient and molecularly compatible materials for fungal bioaerosol capture. Importantly, the results highlight that filter surface chemistry influences capture not only efficiency and viability but also downstream molecular performance. The optimized sampling conditions and validated downstream analysis pipeline provide a robust framework for future applications in environmental mycology, indoor air quality monitoring, and fungal biodiversity assessments.

## Supplementary Information

Below is the link to the electronic supplementary material.

Supplementary file1 (DOCX 3.03 MB)

Supplementary file2 (PDF 847 KB)

## Data Availability

The data that support the findings of this study are avaiable from the corresponding author upon reasonable request.
