# Supplementary material for: Plasma-modified filters improve recovery of viable fungal spores for culture and DNA analysis
Source: Appl Microbiol Biotechnol. 2026 Aug 14;110:342. doi: 10.1007/s00253-026-14006-6 (PMC13751897; doi:10.1007/s00253-026-14006-6)

Plasma-Modified Filters Improve Recovery of Viable Fungal Spores for Culture and DNA Analysis

Authors:

Hafiza Umaima Affan, * [a, b] Claire Lenehan, [a, b] Sally Fryar, [a] Michael Taylor, [a, d,] Melanie MacGregor * [a, c]

Affiliations:

[a] College of Science and Engineering, Flinders University, Sturt Road, Bedford Park, South Australia, 5042, Australia

[b] Flinders Institute for Nanoscale Science and Technology, Flinders University, Sturt Road, Bedford Park, South Australia, 5042, Australia

[c] Nano and Microplastics Research Consortium, Flinders University, Sturt Road, Bedford Park, South Australia, 5042, Australia

[d] WSP Australia Pty Limited, Adelaide, South Australia, 5000, Australia

* Corresponding Author

melanie.macgregor@flinders.edu.au

Supplementary File description

The corresponding uncropped gels images in the manuscript are shown below, with the cropped images marked by red boxes. Along with figure numbers the sample names are also mentioned taken from these gels.

Figure 4a

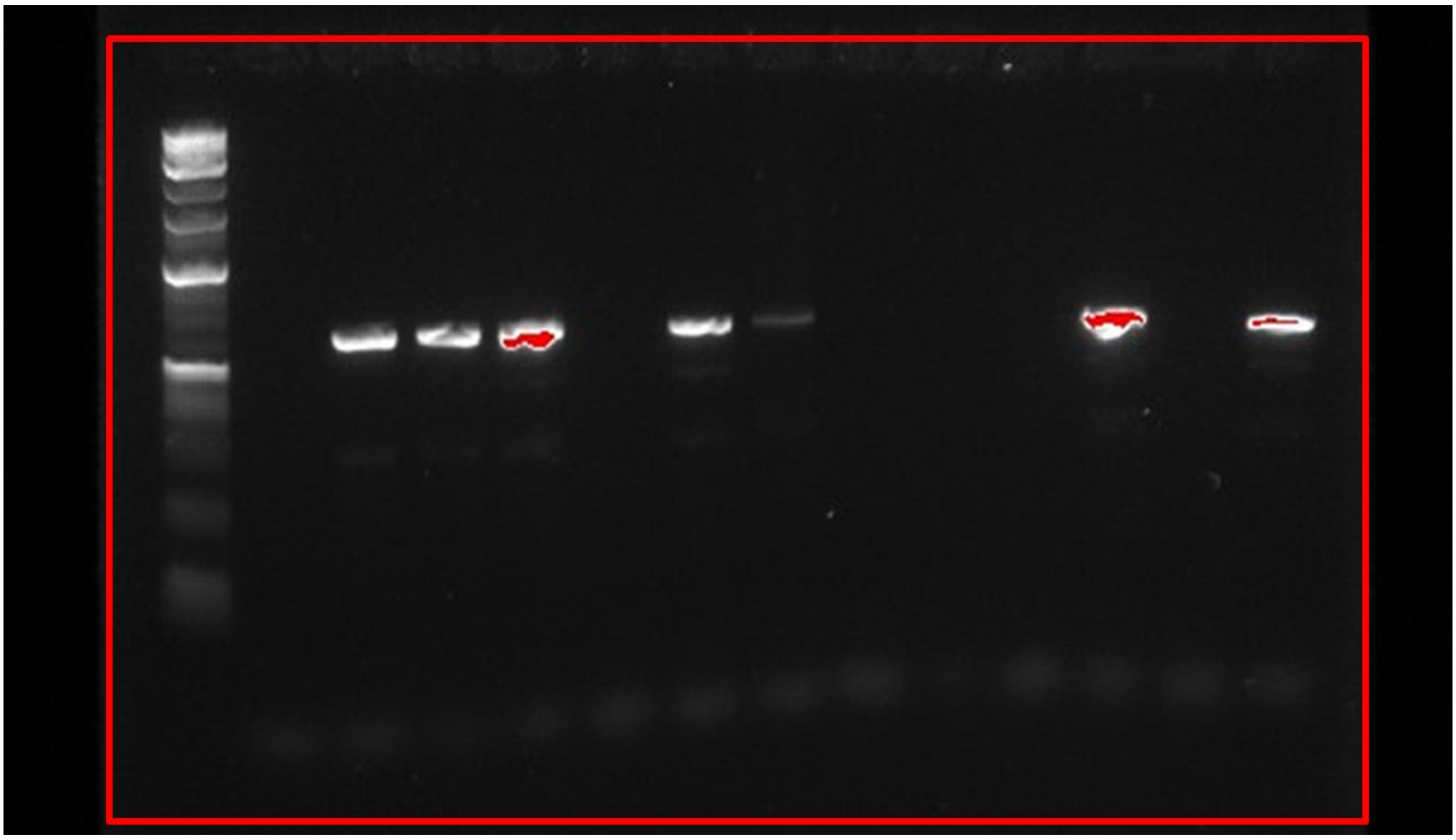

Figure 4b: (LB, -CN, CLP1, CLP2, +CN, LB)

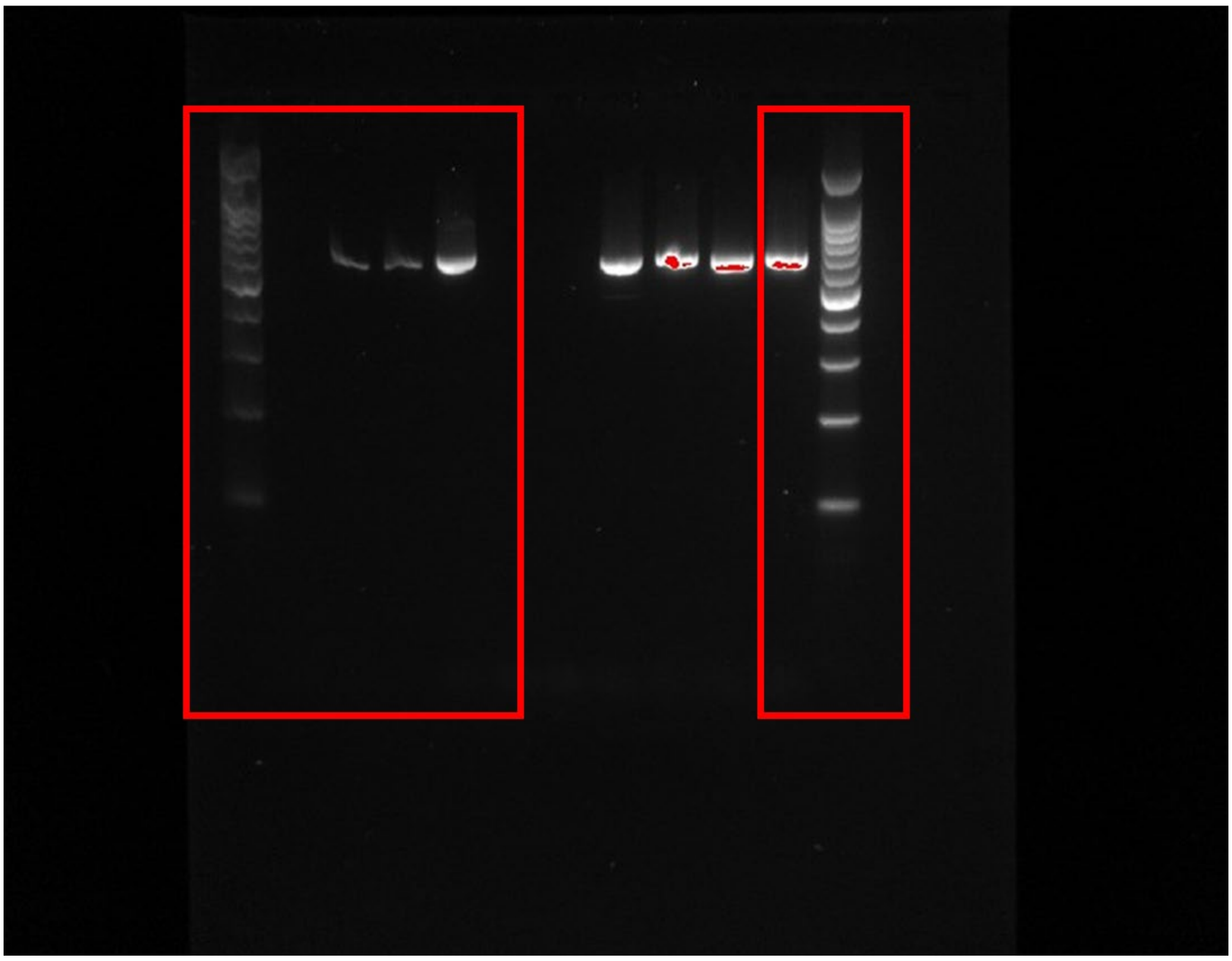

Figure 4b: (CL-F1a, CL-F1b, CL-F1c)

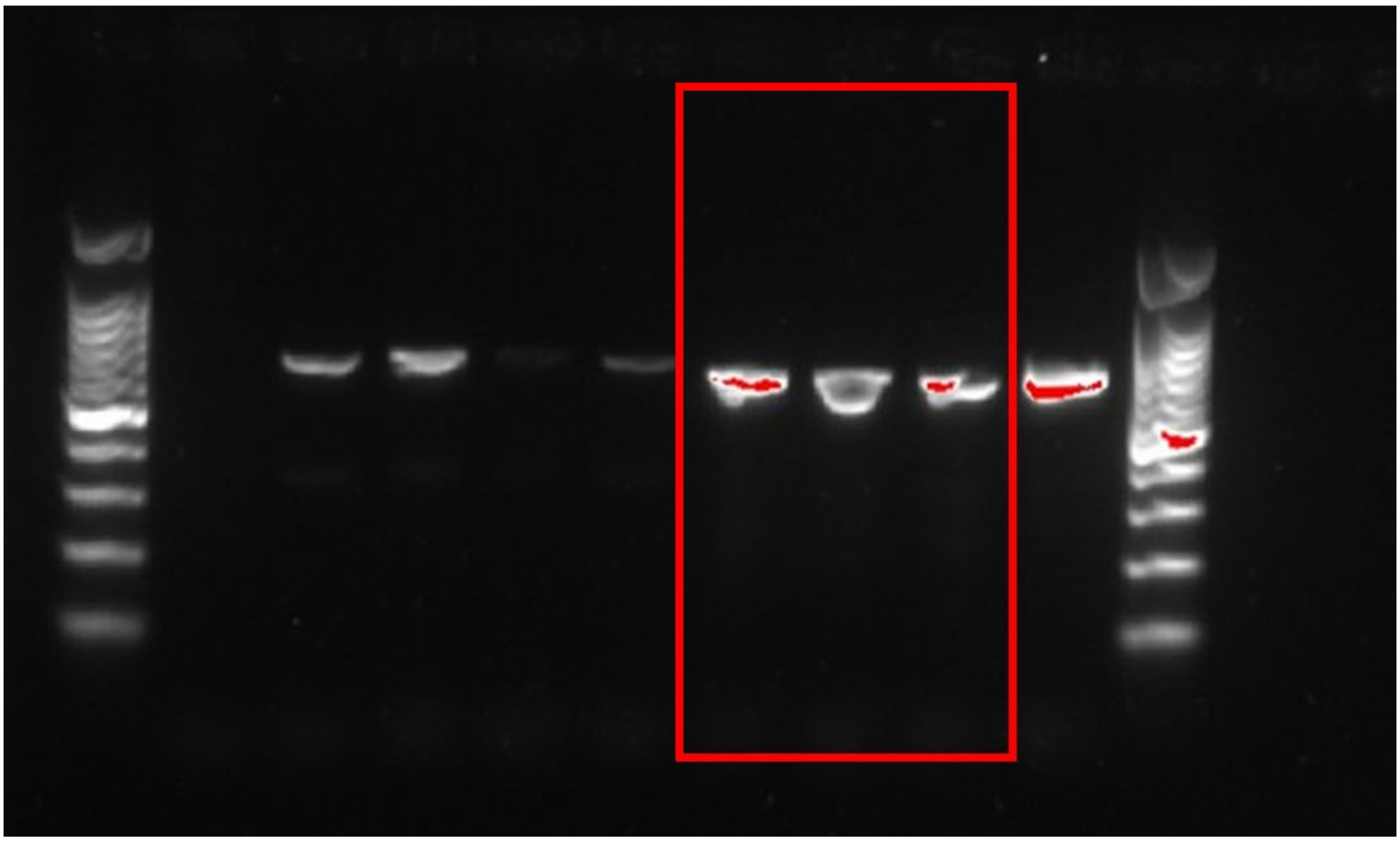

Figure 4b: (CL-F2a, CL-F2b, CL-F2c)

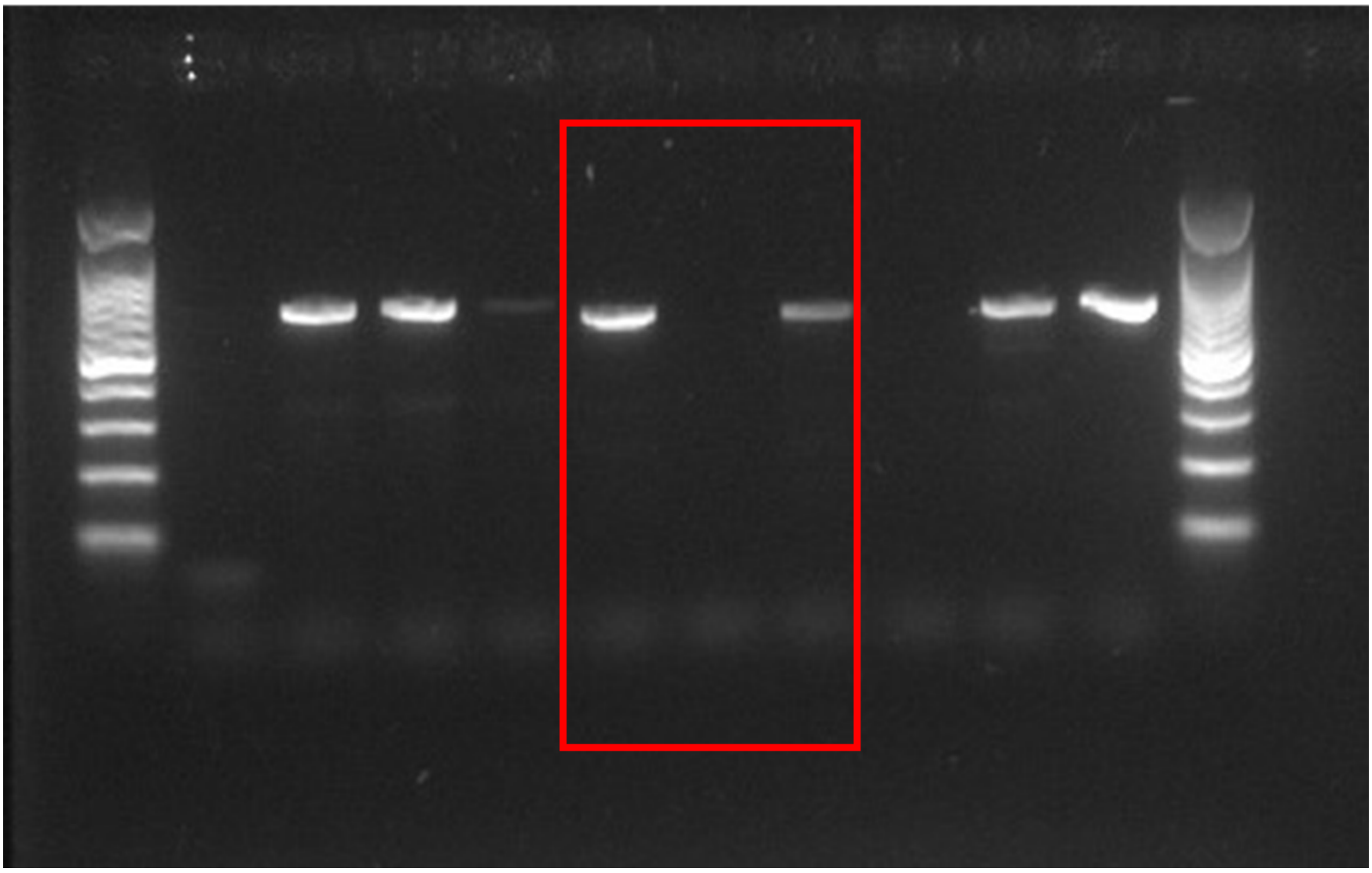

Figure 4b: (CL-F3a, CL-F3b, CL-F3b)

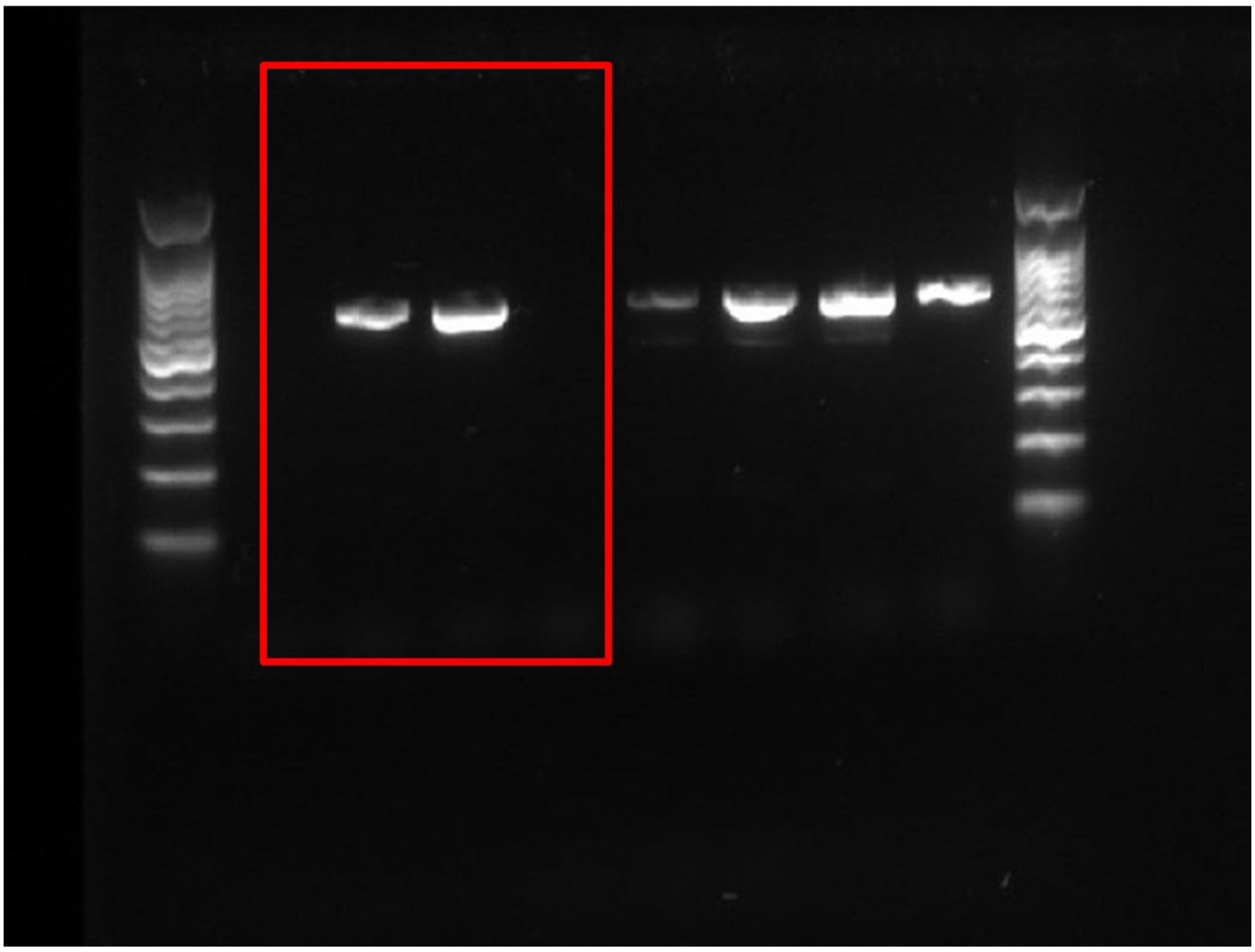

Figure 4c: (LB, -CN, PR-P1, +CN, LB)

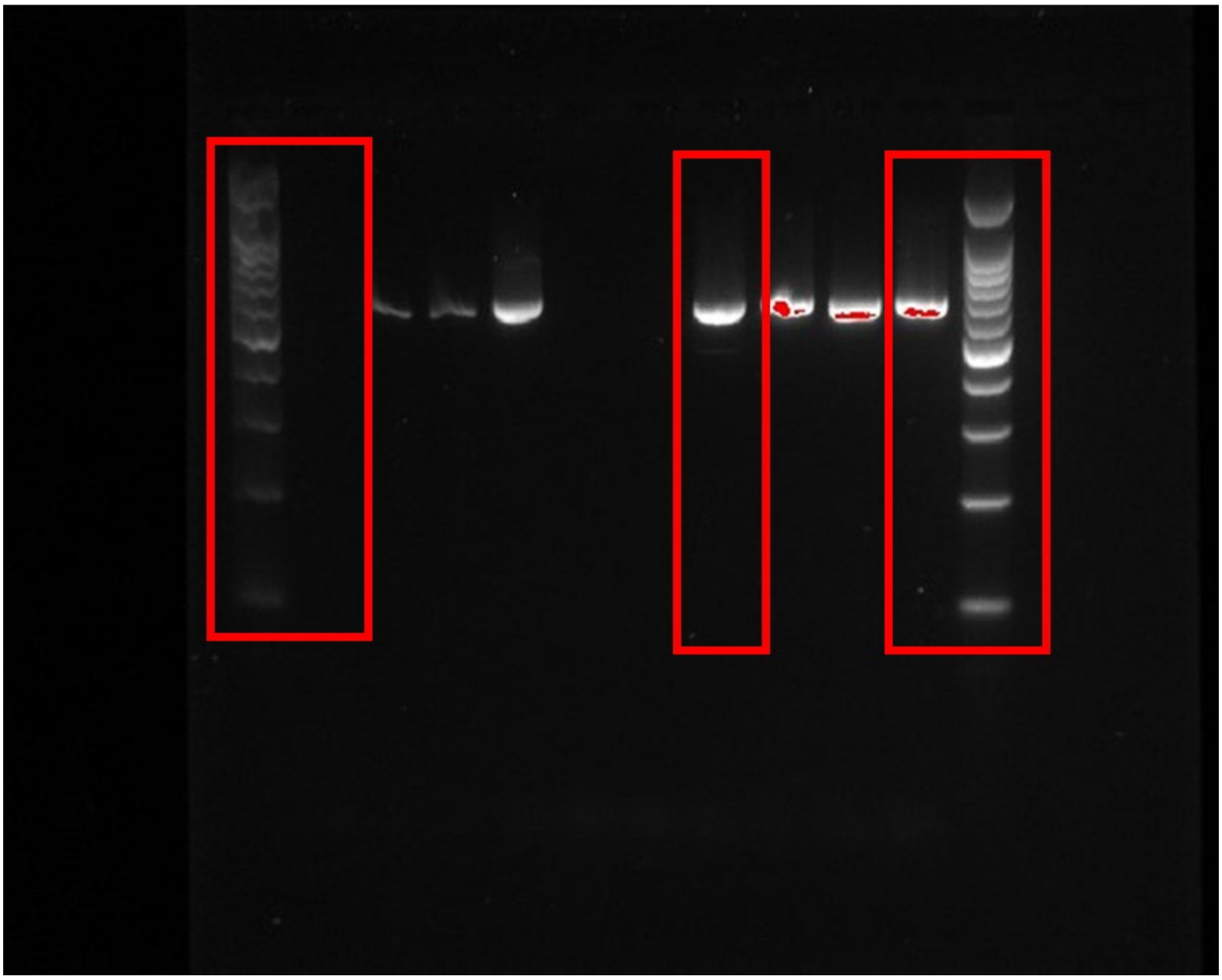

Figure 4c: (Top Rows: PR-P2, PR-F2b, PR-F2c, Bottom row: PRF2a, PR-F1a, PR-F1b, Blank, PR-F2c)

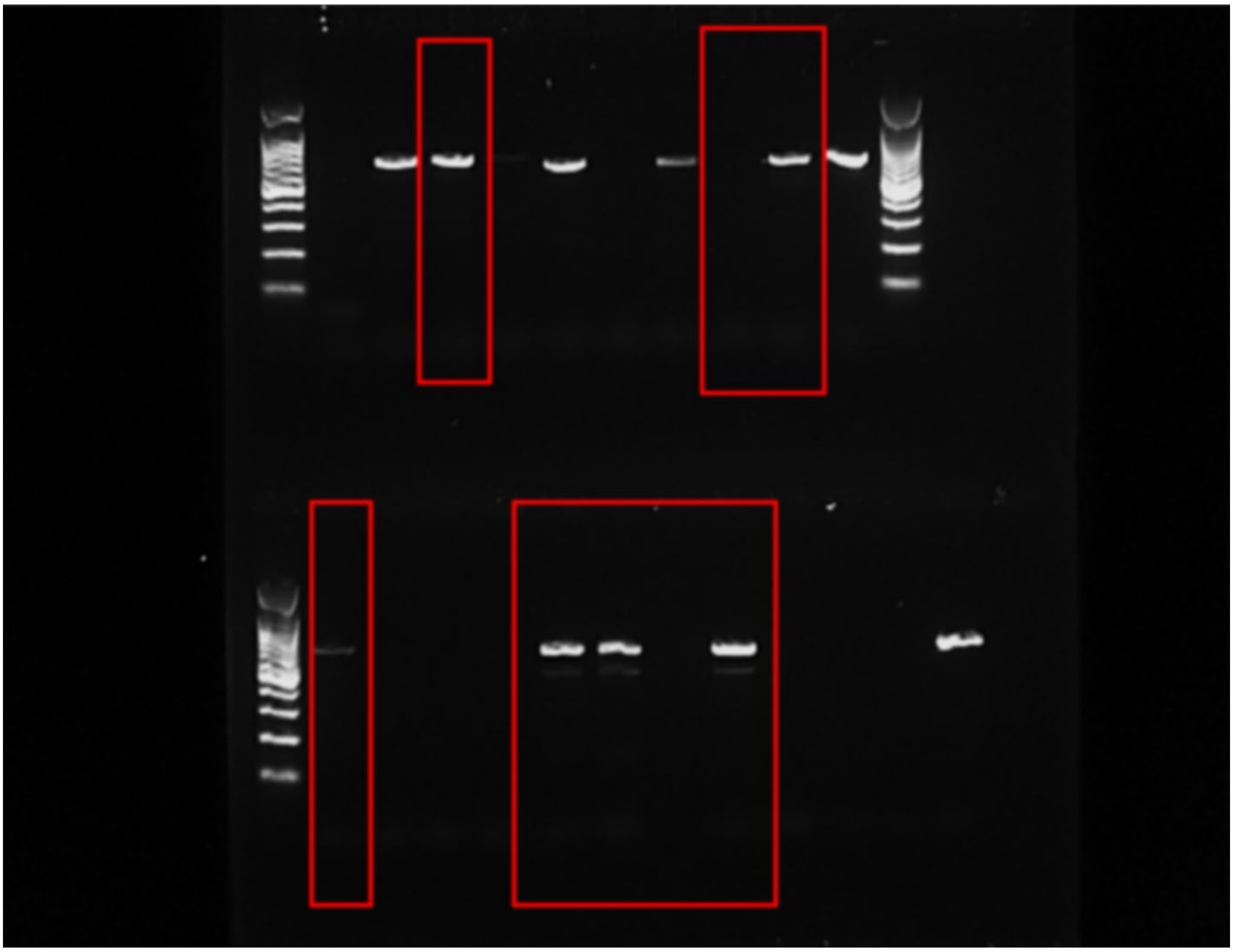

Figure 4c: (PR-F3a, PR-F3b, PR, F3b)

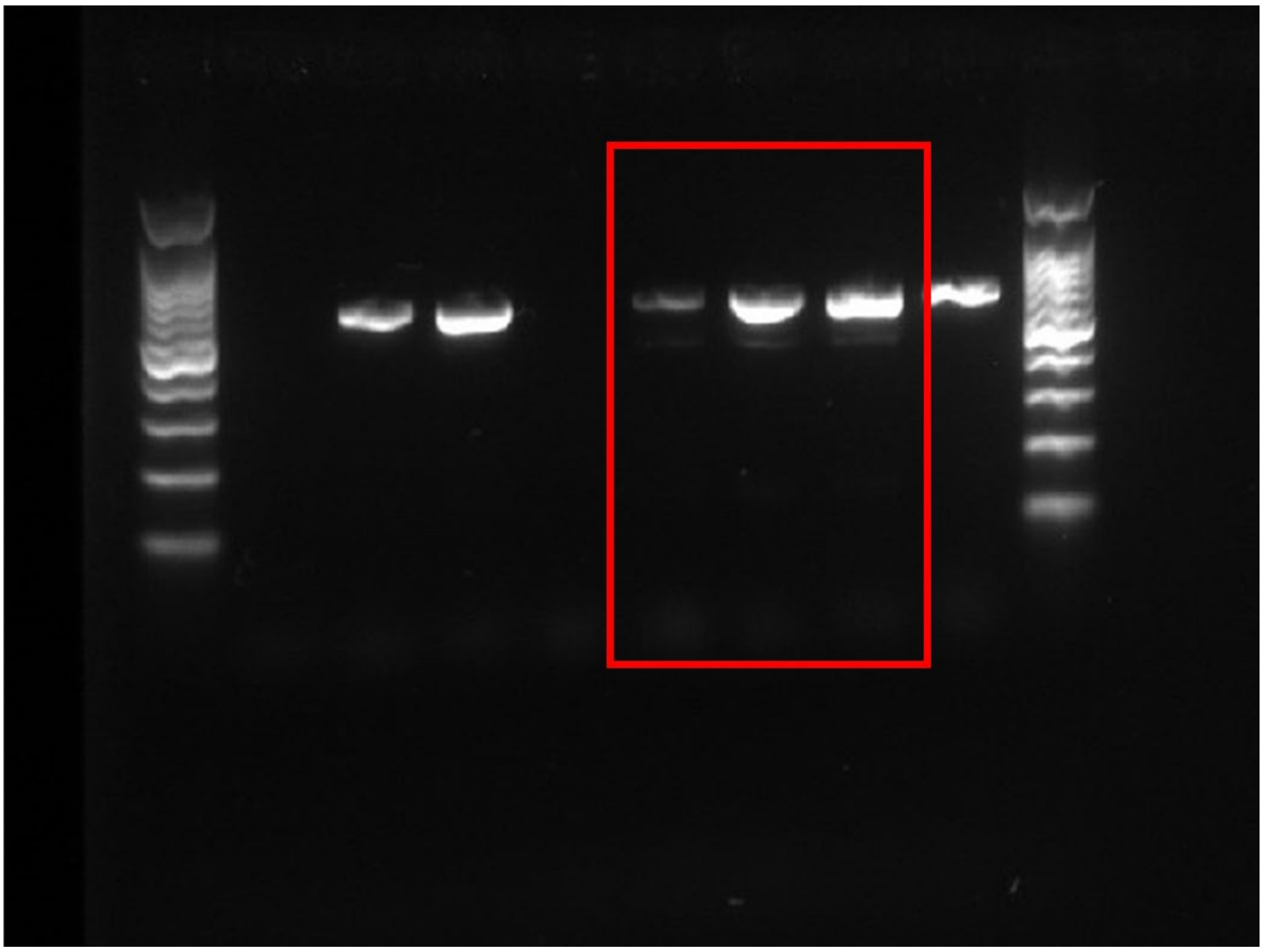

Figure 4d: (LB, -CN, RG-P1, RG-F1a, RG-F1b, +CN, LB)

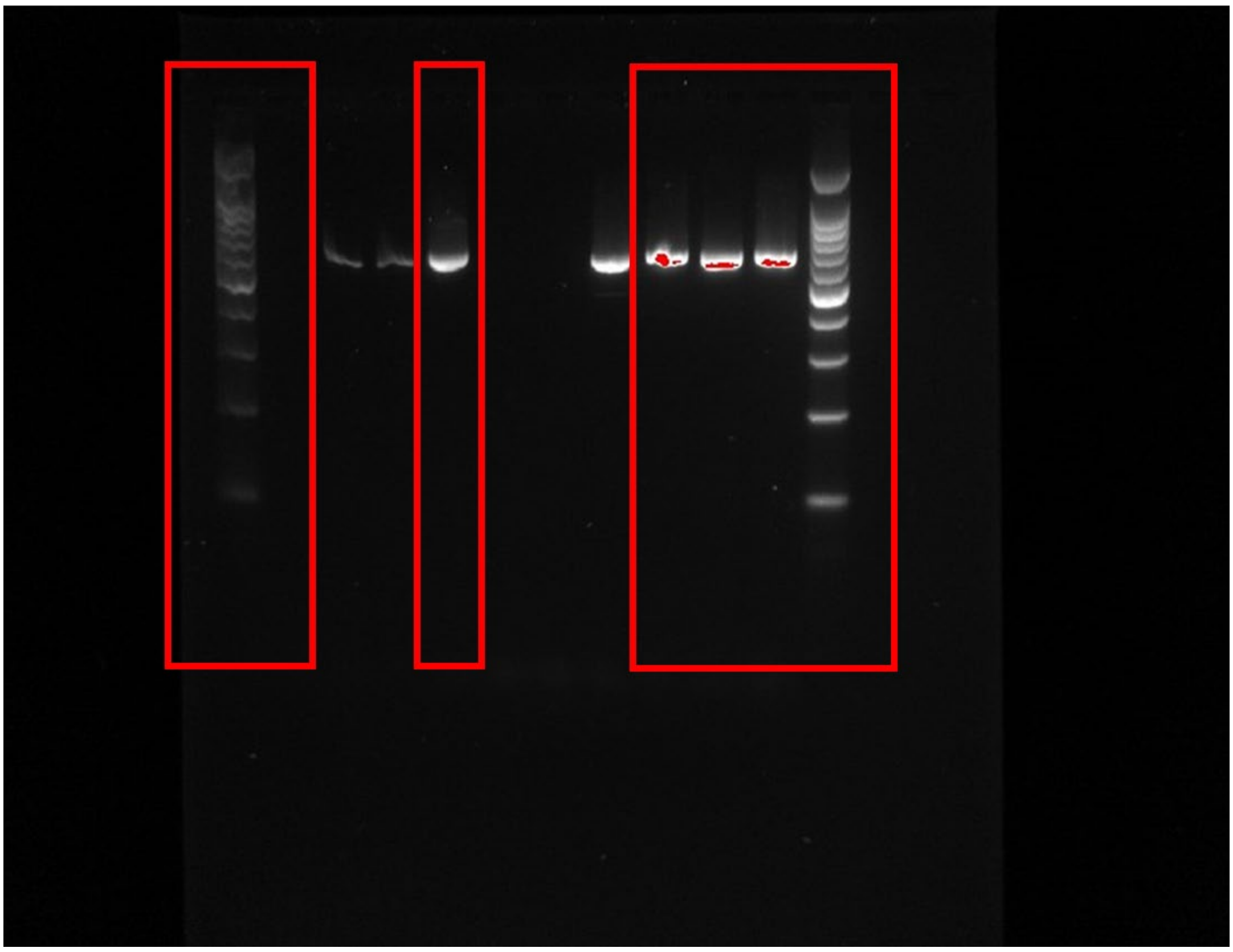

Figure 4d: (RG-P1b)

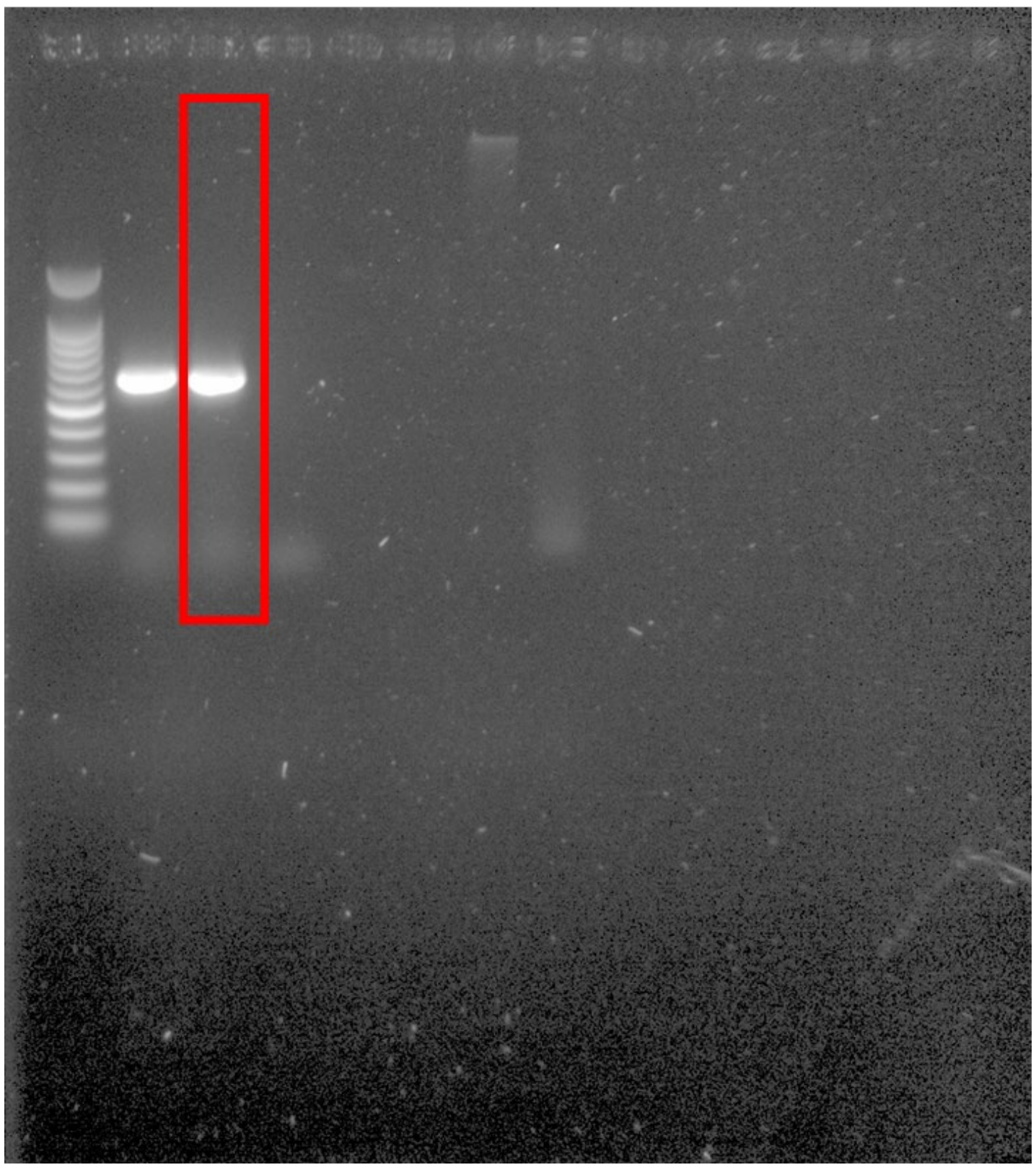

Figure 4d: (RG-F1c, RG-F2a, RG-F2b, RG-F2c)

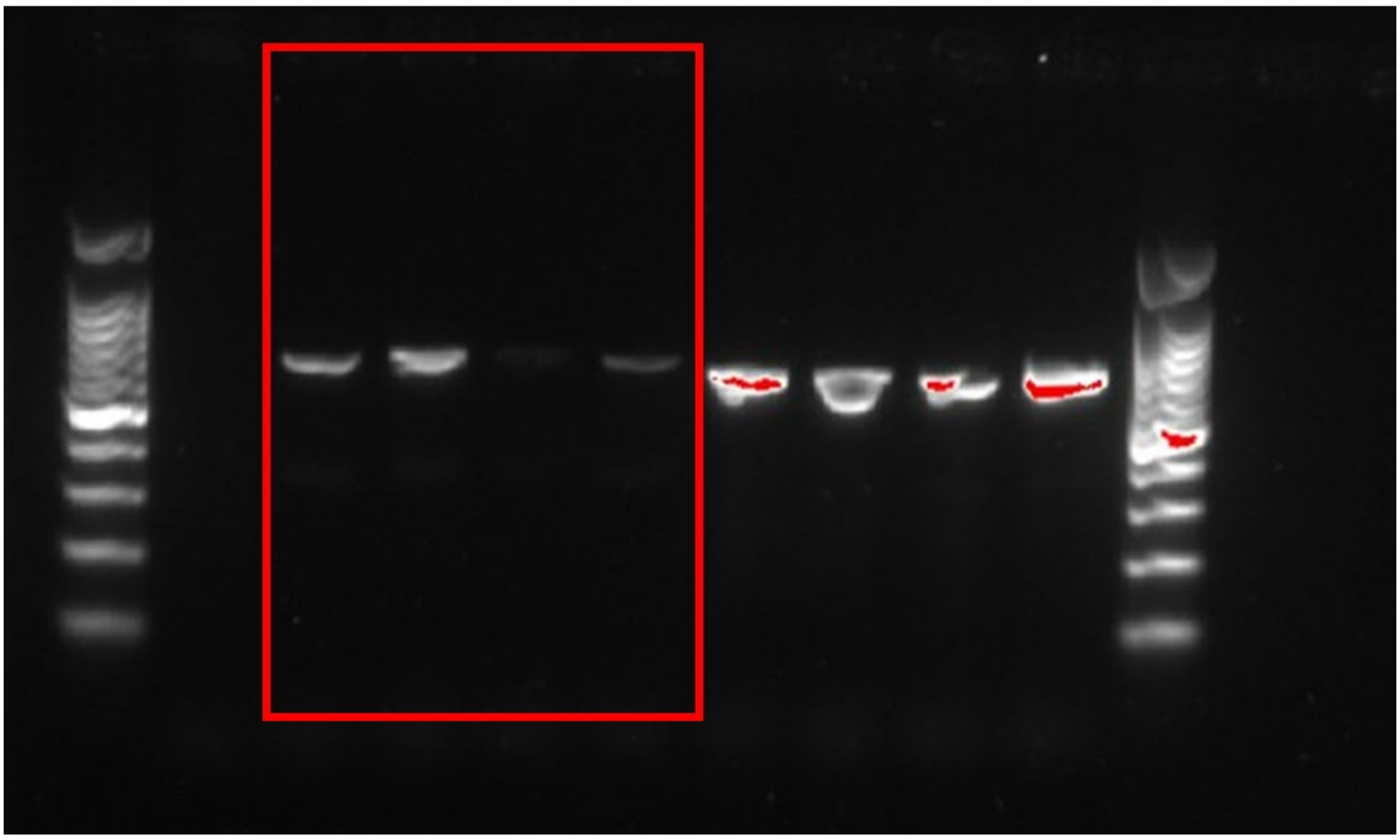

Figure 4d: (RG-F3a, RG-F3b, RG-F3c)

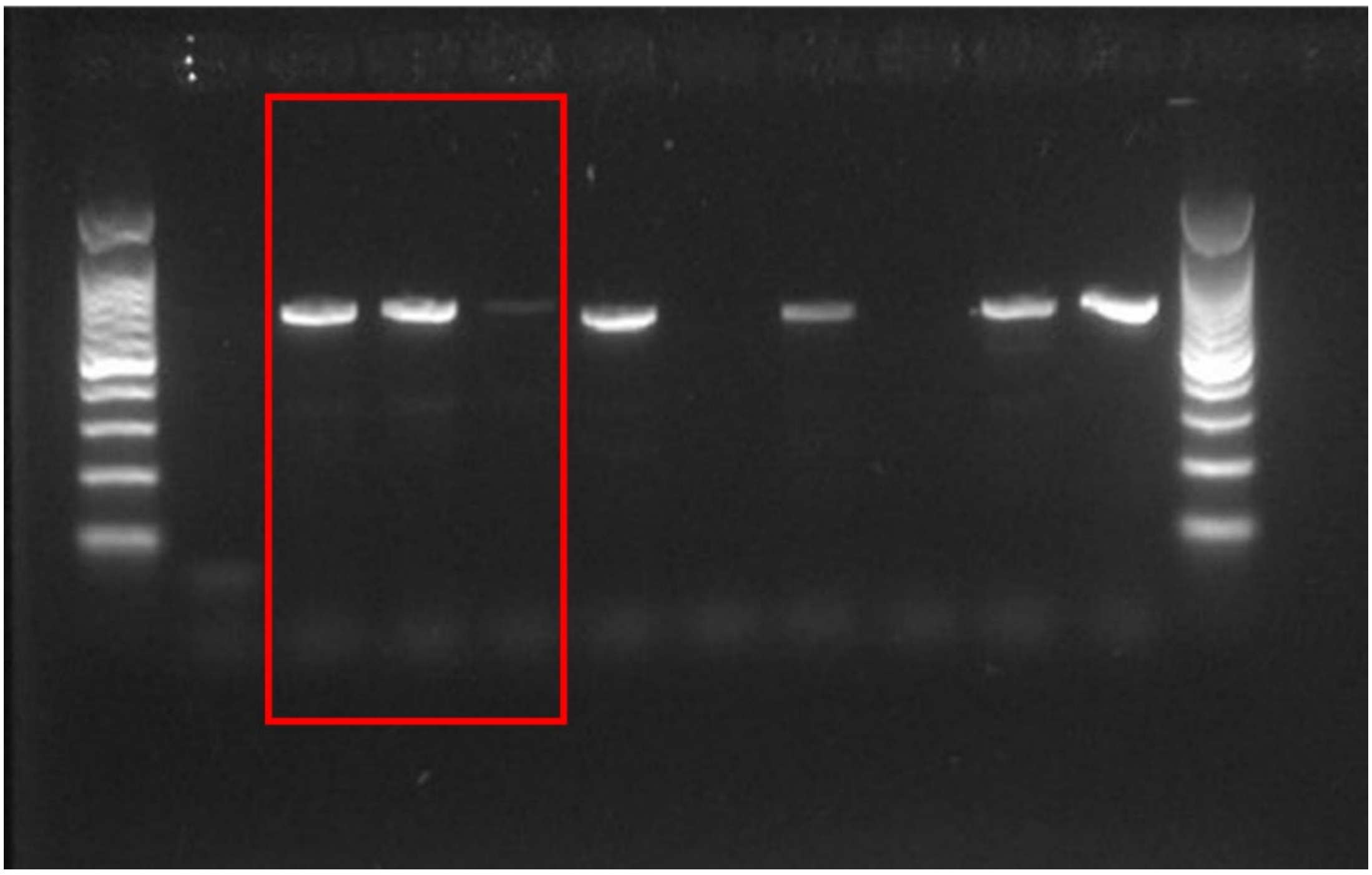

Supplement: Supplementary file 1 [file 253_2026_14006_MOESM2_ESM.pdf]
