# Supplementary material for: Plasma-modified filters improve recovery of viable fungal spores for culture and DNA analysis
Source: Appl Microbiol Biotechnol. 2026 Aug 14;110:342. doi: 10.1007/s00253-026-14006-6 (PMC13751897; doi:10.1007/s00253-026-14006-6)
Supplement: Supplementary file 2 [file 253_2026_14006_MOESM1_ESM.docx]

Applied Microbiology and Biotechnology

**Plasma-Modified Filters Improve Recovery of Viable Fungal Spores for Culture and DNA Analysis**

Hafiza Umaima Affan, * ^a, b^ Claire Lenehan, ^a, b^ Sally Fryar, ^a^ Michael Taylor, ^a, c,^ Melanie MacGregor * ^a, b^

^a^ College of Science and Engineering, Flinders University, Sturt Road, Bedford Park, South Australia, 5042, Australia

^b^ Flinders Institute for Nanoscale Science and Technology, Flinders University, Sturt Road, Bedford Park, South Australia, 5042, Australia

^c^ WSP Australia Pty Limited, Adelaide, South Australia, 5000, Australia

* Corresponding Author

[melanie.macgregor@flinders.edu.au](mailto:melanie.macgregor@flinders.edu.au)

**Supplementary Figures**

**Supplementary Fig. S1** Analysis of different collection times (5, 15, 30, 60, 90 min) on spore collection over non-coated (NC) and plasma coated (POX, PFO) filters from two fungal species (a) Microscopic images of filters captured Aspergillus niger spores (b) Microscopic images of filters captured Rhodotorula glutinis spores. Images taken = 100X magnification. Scale bar = 200 μm.

**Supplementary Fig. S2** Evaluation of non-coated (NC) and plasma coated (POX, PFO) filters used to capture two airborne fungal spores by varying plate numbers (1 - 5 plates). (a) Microscopic images of filters captured Aspergillus niger spores (b) Microscopic images of filters captured Rhodotorula glutinis spores. Images taken = 100X magnification. Scale bar = 200 μm.

**Supplementary Fig. S3** Comparative analysis of saturation behaviour of plasma-coated (POX, PFO) and non-coated (NC) filters using increasing amounts of fungal source material for (a) Aspergillus niger and (b) Rhodotorula glutinis. Data is presented as mean ± standard deviation (SD) from three independent replicates (n = 3). Error bars represent SD; for some results error bars are small and partially obscured by the data markers due to low variability.

**Supplementary Fig. S4** Nucleic acid quantification of fungal biomass from 4 different fungal species using NanoDrop spectrophotometric analysis to determine the minimum biomass required for downstream molecular analyses, including PCR amplification and ITS sequencing. Data is presented as mean ± standard deviation (SD) from 3 individual replicates. AN= Aspergillus niger, CL= Cladosporium sp., PR= Penicillium roqueforti, RG= Rhodotorula glutinis.
